# Supplementary material for: High clonal diversity and spatial genetic admixture in early prostate cancer and surrounding normal tissue
Source: Nat Commun. 2024 Apr 24;15:3475. doi: 10.1038/s41467-024-47664-z (PMC11043350; doi:10.1038/s41467-024-47664-z)
Supplement: Supplementary file 1 — Supplementary Information [file 41467_2024_47664_MOESM1_ESM.pdf]

SUPPLEMENTARY INFORMATION

High clonal diversity and spatial genetic admixture in early prostate cancer and surrounding normal tissue

Ning Zhang, Luuk Harbers, Michele Simonetti, Constantin Diekmann, Quentin Verron, Enrico Berrino, Sara E. Bellomo, Gabriel M.C. Longo, Michael Ratz, Niklas Schultz, Firas Tarish, Peng Su, Bo Han, Wanzhong Wang, Sofia Onorato, Dora Grassini, Roberto Ballarino, Silvia Giordano, Qifeng Yang, Anna Sapino, Jonas Frisén, Kanar Alkass, Henrik Druid, Vassilis Roukos, Thomas Helleday, Caterina Marchiò, Magda Bienko & Nicola Crosetto

1. Supplementary Figures pg. 2
2. Supplementary Methods pg. 45
3. Supplementary Tables pg. 51
4. Supplementary References pg. 52

1. Supplementary Figures

Supplementary Figure 1

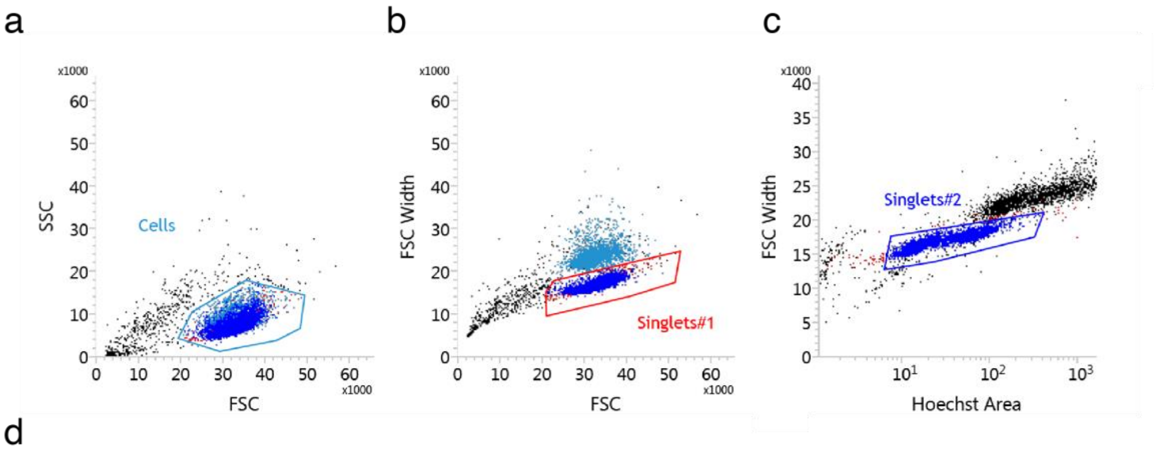

d

Populations	Even...	% Total	% Parent
All Events	7,008	100.00%	####
Cells	6,462	92.21%	92.21%
Singlets#1	4,246	60.59%	65.71%
Singlets#2	4,152	59.25%	97.79%

Supplementary Fig. 1. Gating strategy used to sort cells or nuclei stained with the DNA intercalator, Hoechst 33342 (see **Methods**). The data plotted in (**a-c**) were obtained in a sorting experiment of fixed SKBR3 cells, which we initially performed to set up sorting parameters for all subsequent experiments. The table in (**d**) summarizes the fraction of events in the corresponding gates in (**a-c**). The plots were generated using the BD FACSJazz Cell Sorter control software at the Biomedicum Flow cytometry Core facility (Karolinska Institutet), supported by KI/SLL. SSC, side scattering. FCS, forward scattering.

Supplementary Figure 2

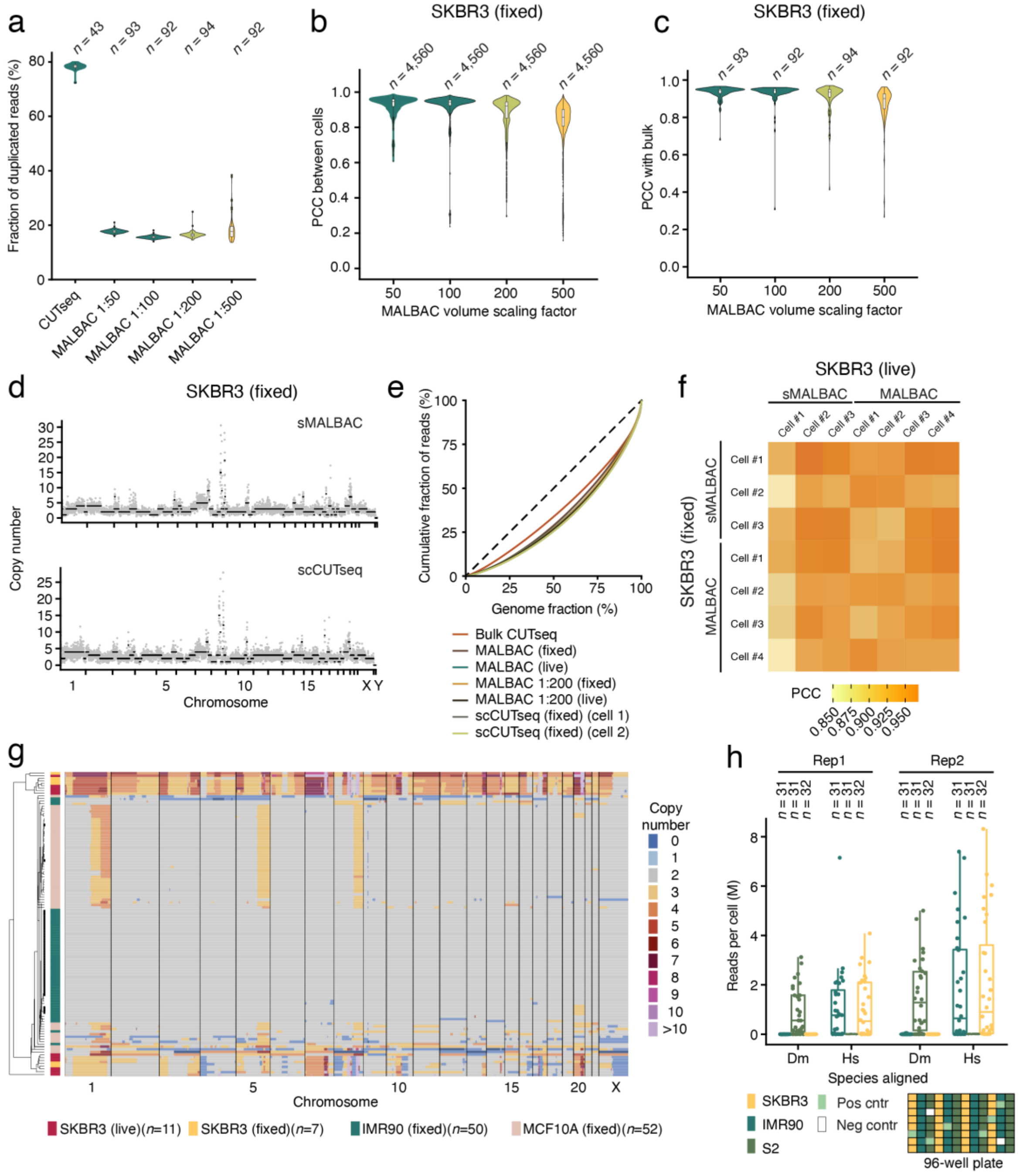

Supplementary Fig. 2. Technical performance and reproducibility of scCUTseq. (**a**) Fraction of read duplicates obtained after performing standard CUTseq directly on single cells or performing CUTseq after MALBAC by scaling reagent volumes 50, 100, 200 and 500 times. *n*, number of single cells with over 50K reads (**b**) Pearson's correlation coefficient (PCC) for all possible pair-wise comparisons between the segmented copy number profiles of individual SKBR3 cells processed by scCUTseq, using different MALBAC reagent volume scaling

factors. *n*, number of pair-wise comparisons in each group. (**c**) As in (b) but comparing the copy number profile of each single cell with the copy number profile of the corresponding cell line determined by bulk CUTseq. *n*, number of cells compared to bulk CUTseq. In (a-c), violins extend from minimum to maximum, each box in the boxplot inside each violin spans from the 25th to the 75th percentile and whiskers extend from –1.5×IQR to +1.5×IQR from the closest quartile, where IQR is the inter-quartile range. Black dots, outliers. (**d**) Example of single-cell copy number profiles of fixed SKBR3 cells determined by performing a 1:200 scaled-down version of MALBAC (sMALBAC) followed either by standard library preparation or by CUTseq (scCUTseq). Each gray dot represents a 500 kilobases (kb) genomic bin. Black dots indicate segmented copy number profiles determined by circular binary segmentation. (**e**) Lorenz curves for genomic coverage by bulk CUTseq, standard MALBAC, 1:200 sMALBAC, and scCUTseq on SKBR3 cells. (**f**) Correlation matrix showing the similarity between the segmented copy number profiles of individual fixed or non-fixed (live) SKBR3 cells obtained by performing standard MALBAC or 1:200 sMALBAC followed by library preparation with a commercial kit (NEBNext, see **Methods**). (**g**) Hierarchically clustered single-cell copy number profiles obtained by applying scCUTseq to four different cell lines, with or without mild fixation (4% PFA for 10 min, see **Methods**). *n*, number of single cells. (**h**) Distributions of scCUTseq reads per cell after alignment to the *Homo sapiens* (Hs) or *Drosophila melanogaster* (Dm) reference genomes for three different cell types (Hs IMR90 and SKBR3 cells; Dm S2 cells) sorted in different columns of a 96-well plate as shown in the bottom scheme. Pos cntr, positive control consisting of 20 pg of genomic DNA from each cell line used as input for scCUTseq. Neg cntr, negative control consisting of nuclease-free water. *n*, number of cells analyzed. In all boxplots, each box spans from the 25th to the 75th percentile and whiskers extend from –1.5×IQR to +1.5×IQR from the closest quartile, where IQR is the inter-quartile range. Each dot corresponds to single well in the plate scheme shown below. A link to the Source Data for this figure is provided in the Data Availability statement.

Supplementary Figure 3

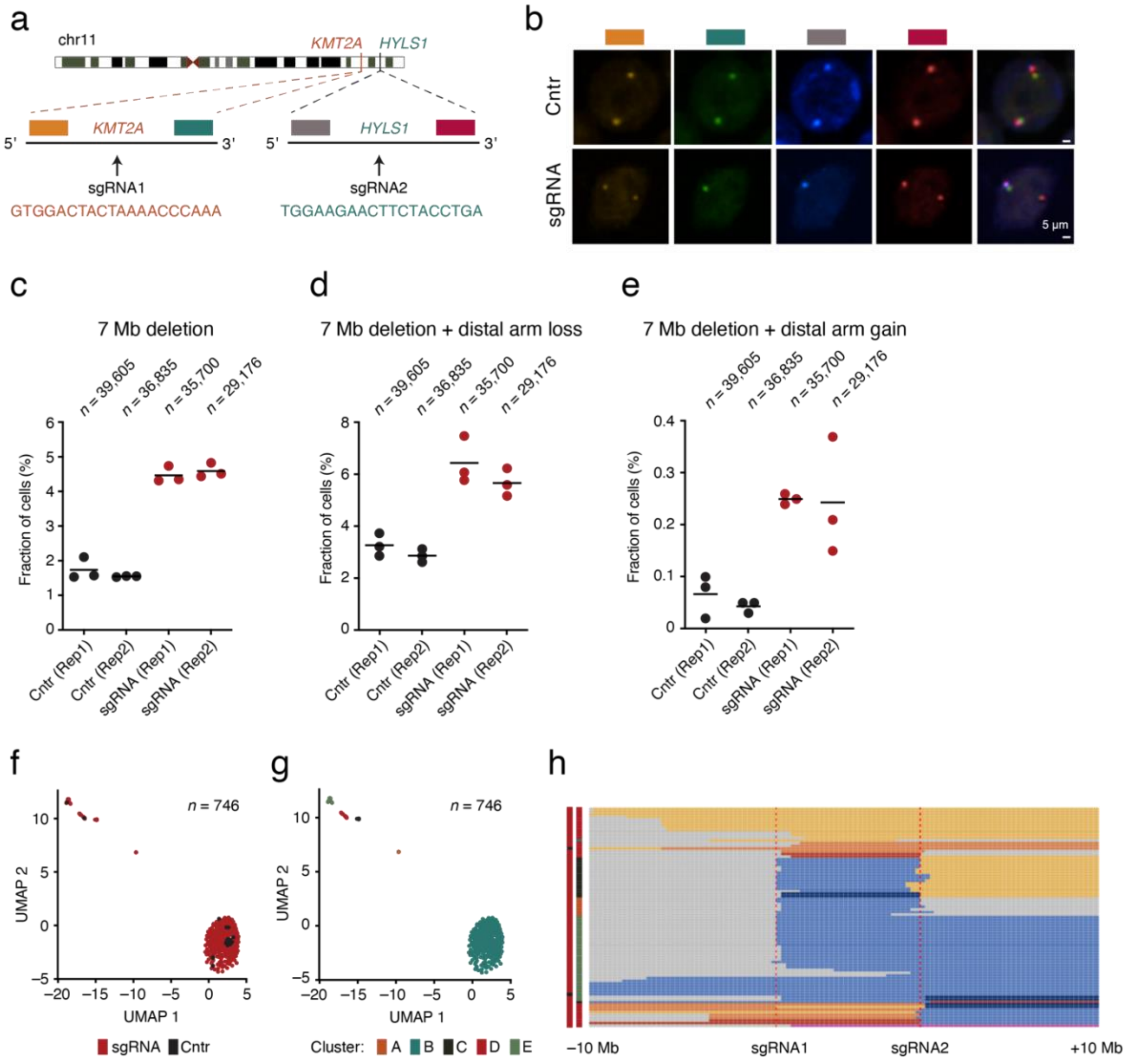

Supplementary Fig. 3. scCUTseq sensitivity assessment. (**a**) Scheme of DNA fluorescence in situ hybridization (FISH) probes (colored rectangular bars) surrounding the *KMT2A* and *HYLS1* gene loci on chr11, used to detect a 7 Mb deletion induced by CRISPR-Cas9 using the two displayed small-guide RNAs (sgRNA). (**b**) Visualization of the FISH probes shown in (a) in human TK6 cells either transfected with non-targeting sgRNA (Cntr) or with both sgRNAs shown in (a) (sgRNA). The colored bars on the top correspond to the FISH probes shown in (a). In sgRNA treated cells in which the correct 7 Mb deletion event has occurred, the probes downstream of the *KMT2A* locus (green) and upstream of the *HYLS1* locus (grey) are detected only on one chr11 homologue, as expected. Scale bars, 5 µm. (**c**) Fraction of TK6 cells carrying the exact 7 Mb deletion on chr11. Each dot represents the fraction of cells containing the

chromosomal rearrangement from a FISH technical replicate (one out of three wells of a 96-well plate). Horizontal black bars represent the mean. Rep, biological replicates. (**d**) As in (c), but for cells in which the 3′ portion of chr11 after the 7 Mb deletion was lost. (**e**) As in (c), but for cells in which the 3′ portion of chr11 after the 7 Mb deletion was amplified. (**f**) Dimensionality reduction by Uniform Manifold Approximation and Projection (UMAP) of copy number profiles of edited (sgRNA) and non-edited (Cntr) TK6 cells. Each dot represents a single cell. *n*, number of single cells. (**g**) As in (f), but with different clusters identified shown in different colors. (**h**) Single-cell copy number profiles (250 kb resolution) of TK6 cells harboring copy number alterations at the targeted location on chr11. The color bars indicate edited (sgRNA) and non-edited (Cntrl) TK6 cells and different UMAP clusters, as in (f) and (g), respectively. A link to the Source Data for this figure is provided in the Data Availability statement.

Supplementary Figure 4

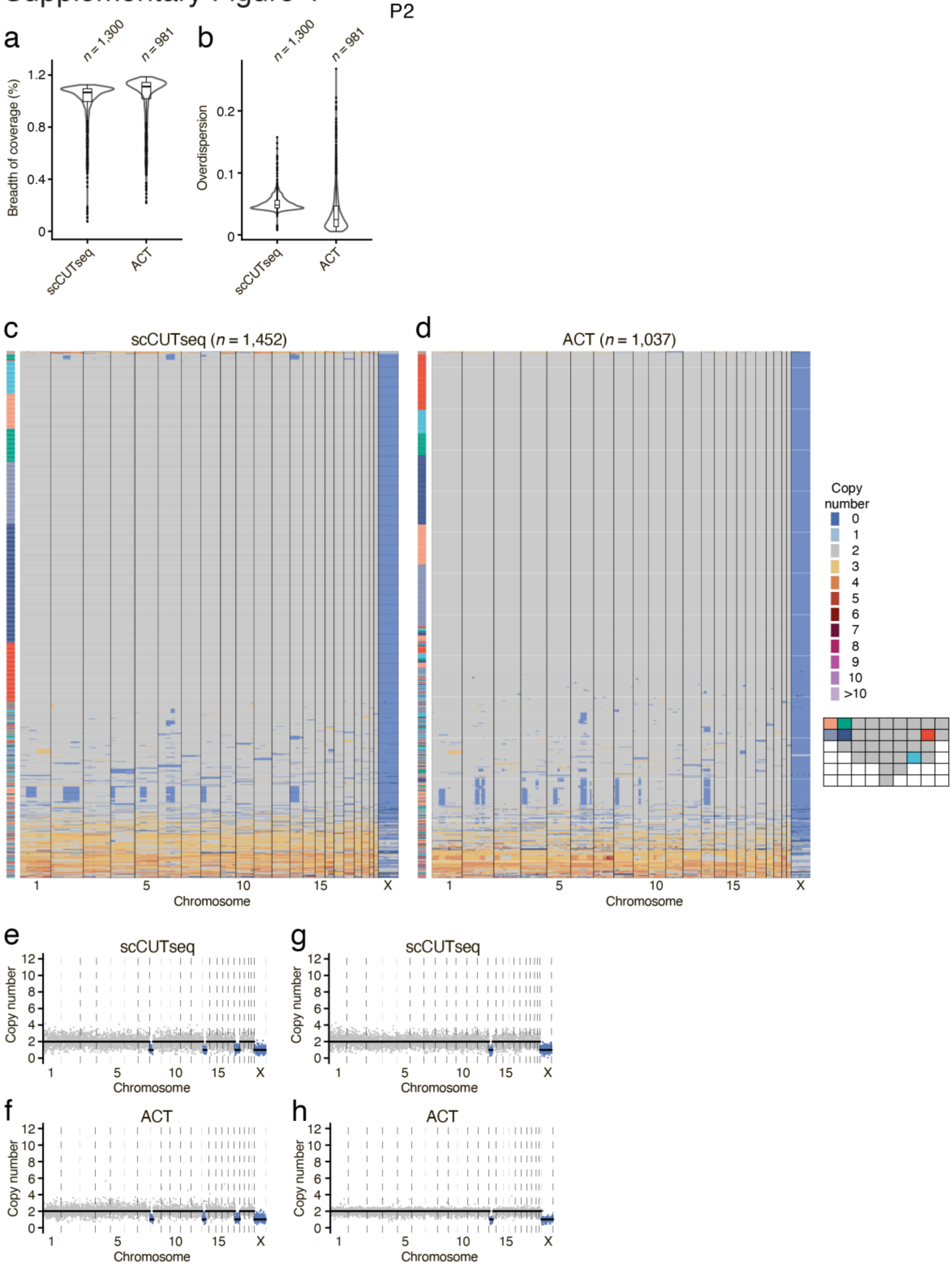

Supplementary Fig. 4. scCUTseq benchmarking. (**a**) Distributions of the breadth of genome coverage obtained with scCUTseq vs. Acoustic Cell Tagmentation (ACT) for the indicated number (*n*) of nuclei extracted from six different regions in prostate sample P2 (see scheme on

the bottom right in (d). Violins extend from minimum to maximum, each box in the boxplot inside each violin spans from the 25th to the 75th percentile and whiskers extend from –1.5×IQR to +1.5×IQR from the closest quartile, where IQR is the inter-quartile range. Black dots, outliers. (**b**) As in (a) but for the overdispersion of binned read counts along the genome calculated as described in the Methods. (**c, d**) Single-cell copy number profiles (500 kb resolution) of the same cells profiled by scCUTseq (c) or ACT (d) analyzed in (a) and (b). The rectangular scheme on the bottom right in (d) shows the regions in prostate sample P2 from which the nuclei profiled by scCUTseq and ACT were obtained. See **Fig. 1c** for the corresponding histopathologic annotation. (**e-h**) Examples of copy number profiles (500 kb resolution) of pseudo-diploid cells displaying the same deletion patterns detected by both scCUTseq and ACT (compare (e) with (f) and (g) with (h)). Each plot corresponds to one cell. A link to the Source Data for this figure is provided in the Data Availability statement.

Supplementary Figure 5

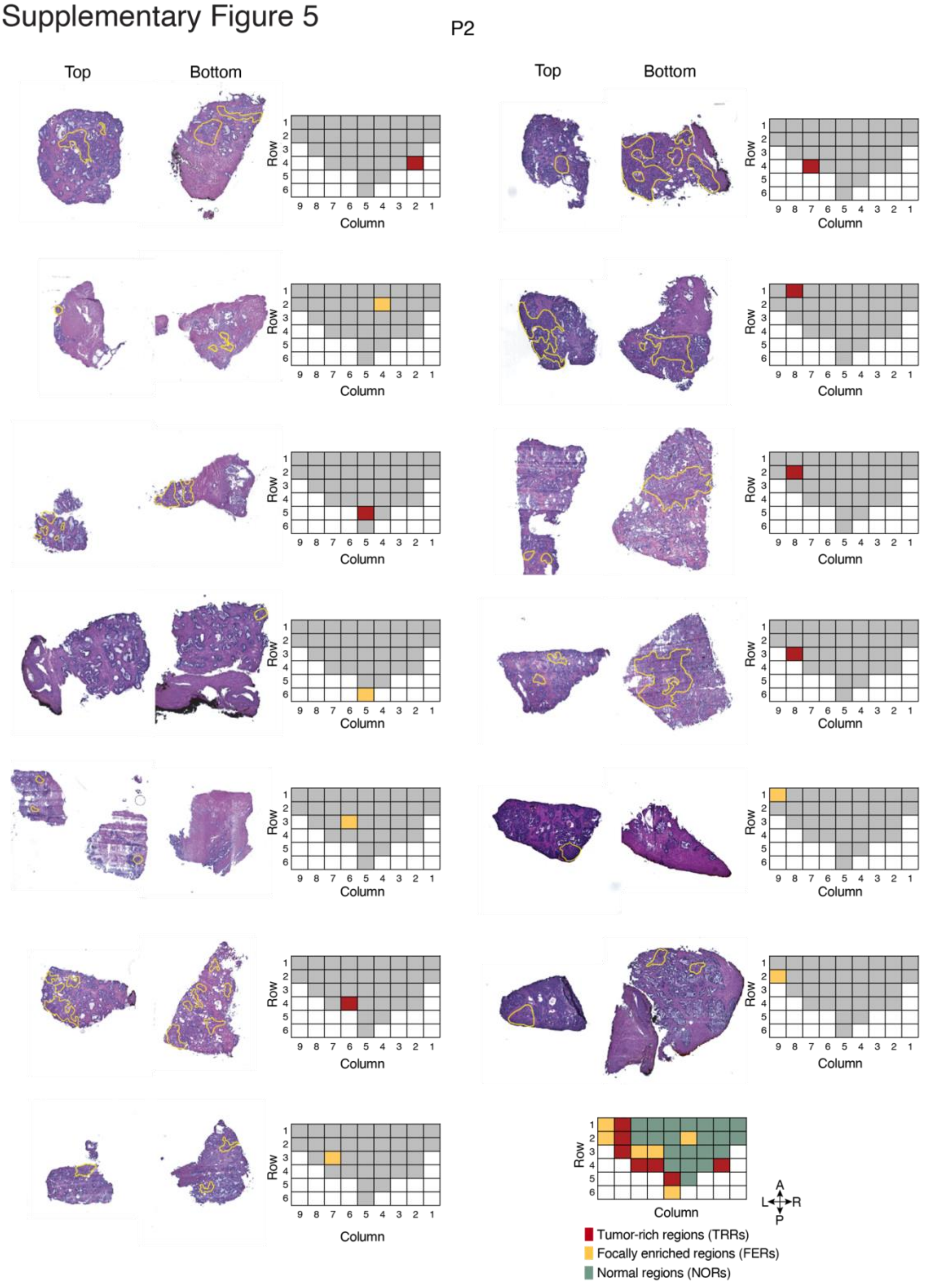

Supplementary Fig. 5. Pathological annotation of hematoxylin-eosin (H&E) stained tissue sections cut from the top and the bottom of each tissue cube in prostate sample P2. Tumor regions are delineated by the yellow lines on top of each H&E-stained section. For each pair of

sections, the position of the corresponding tissue cube (region) is depicted on the map on the right. Only regions containing tumor cells are shown. The map on the bottom right is as the one shown in **Fig. 1c**. Two board-certified pathologists examined the sections independently and classified them based on the percentage of tumor cells. A link to the Source Data for this figure is provided in the Data Availability statement.

Supplementary Figure 6

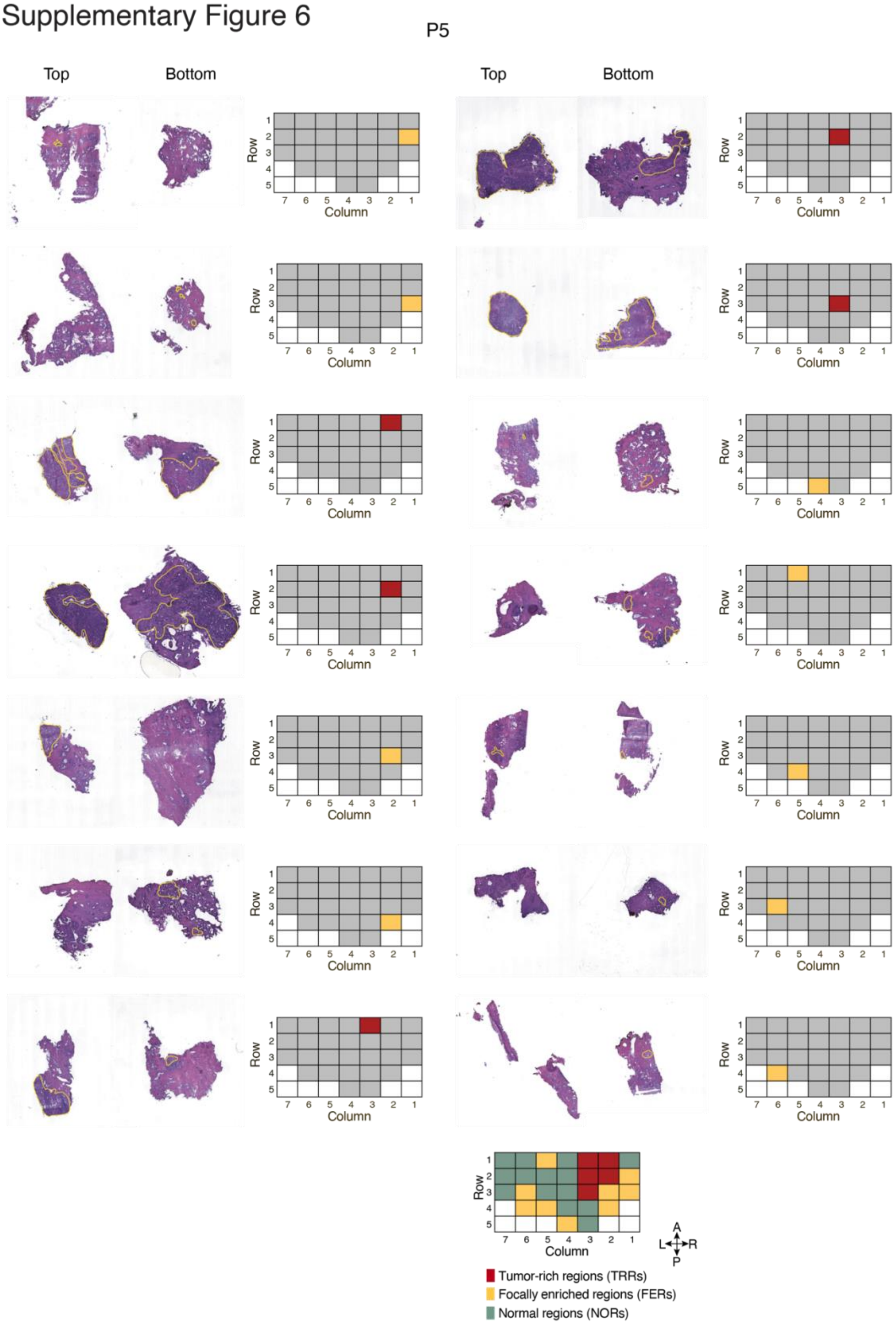

Supplementary Fig. 6. Pathological annotation of hematoxylin-eosin (H&E) stained tissue sections cut from the top and the bottom of each tissue cube in prostate sample P5. Tumor

regions are delineated by the yellow lines on top of each H&E-stained section. For each pair of sections, the position of the corresponding tissue cube (region) is depicted on the map on the right. Only regions containing tumor cells are shown. The map on the bottom right is as the one shown in **Fig. 1d**. Two board-certified pathologists examined the sections independently and classified them based on the percentage of tumor cells. A link to the Source Data for this figure is provided in the Data Availability statement.

Supplementary Figure 7

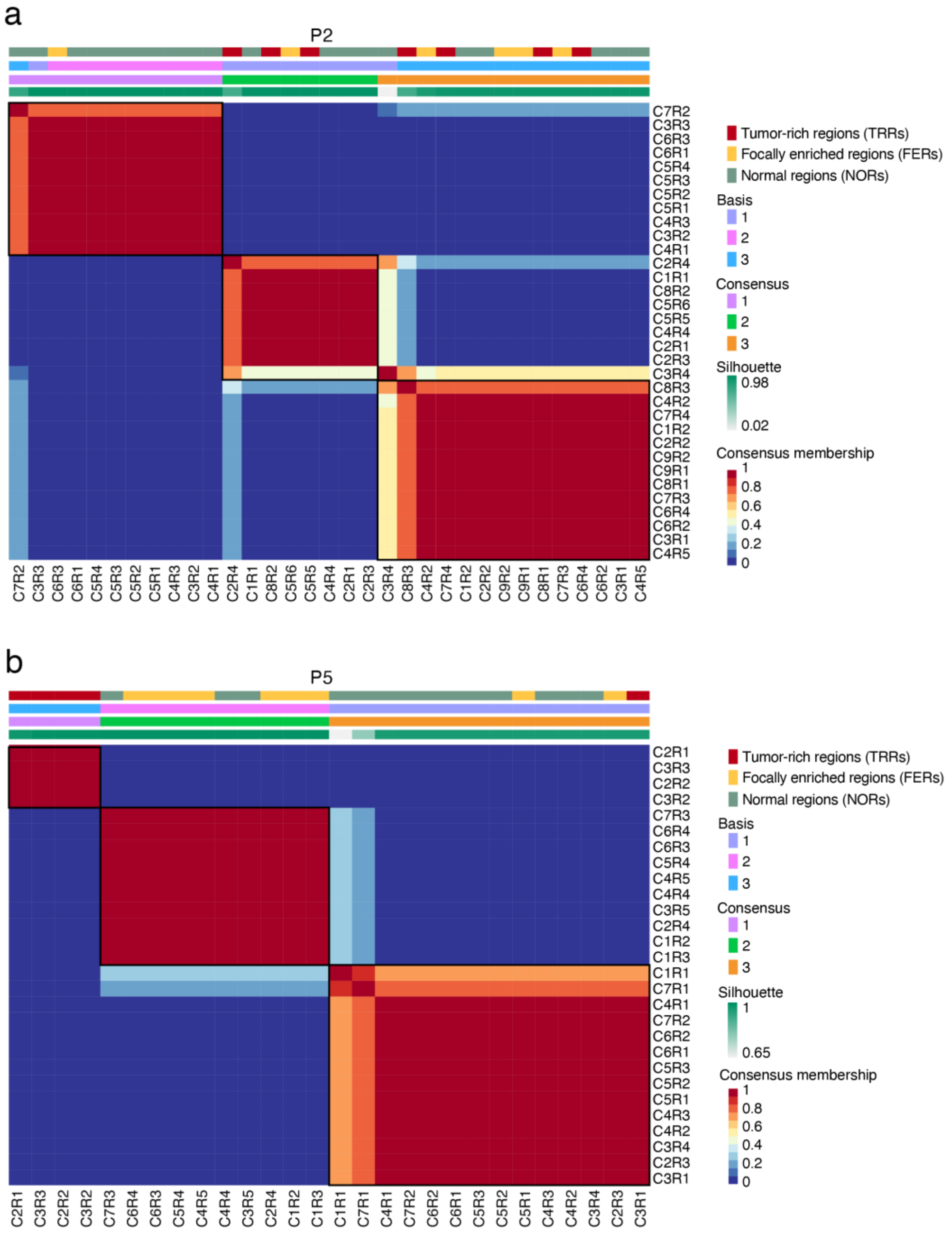

Supplementary Fig. 7. Annotation by RNA-seq of tissue regions in prostate samples P2 and P5. See **Supplementary Methods** for how RNA-seq data clustering was performed. (**a**) Quality of clustering of the regions in P2 for *n*=3 clusters corresponding to the number of different region types defined based on histopathology. The position of each region in the corresponding tissue map in **Fig. 1c** is indicated (C, column; R, row). (**b**) As in (a) but for sample P5. The

position of each region in the corresponding tissue map in **Fig. 1d** is indicated. A link to the Source Data for this figure is provided in the Data Availability statement.

Supplementary Figure 8

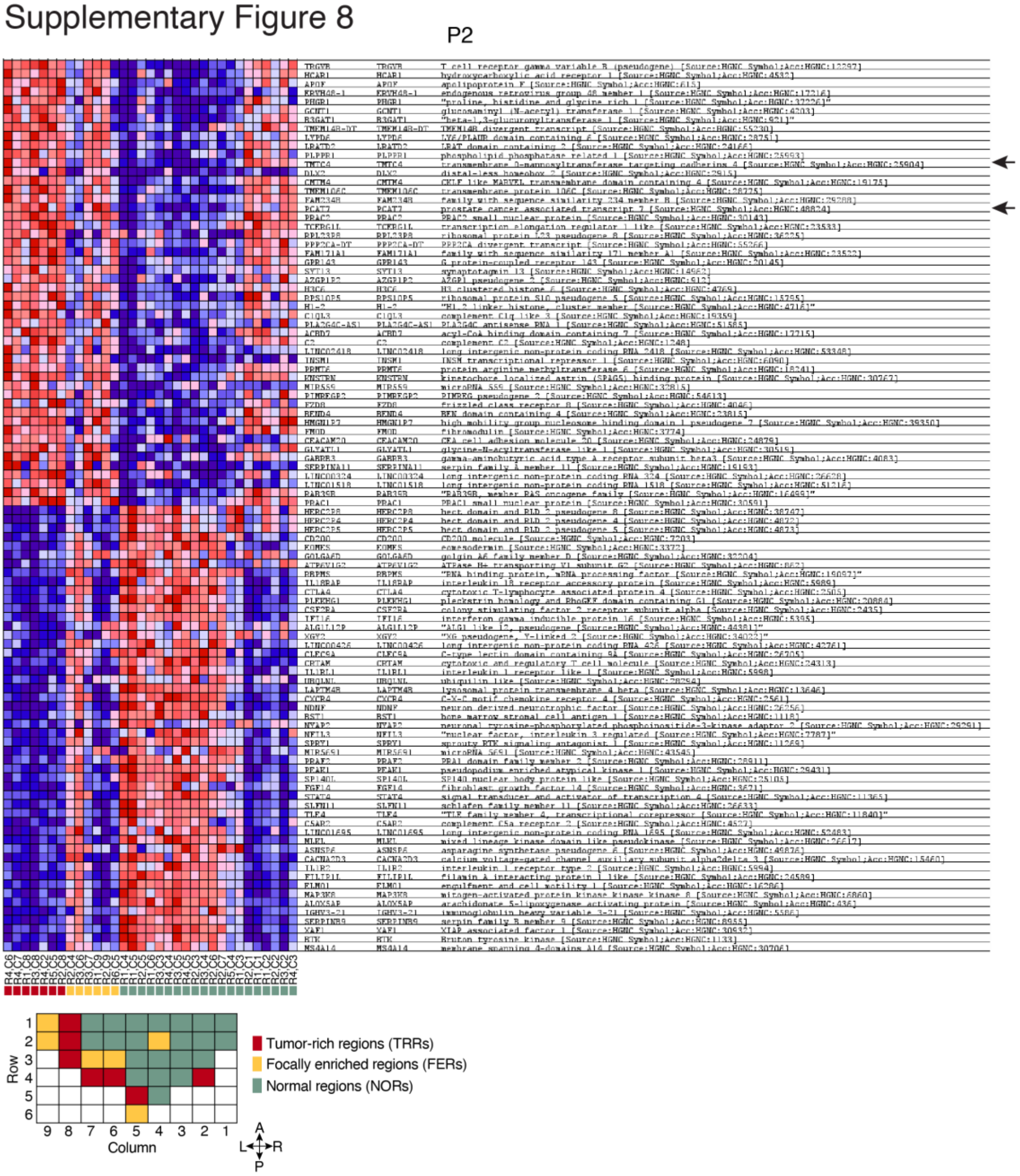

Supplementary Fig. 8. Gene set enrichment analysis (GSEA) comparing tumor-rich regions (TRRs) versus focally enriched regions (FERs) and normal regions (NORs) in prostate sample P2. The top 50 up- and down-regulated genes are shown. The arrows on the right indicate two prostate cancer biomarker genes (*PCA3* and *TMTC4*) that are upregulated in TRRs. The tissue map on the bottom left is as in **Fig. 1c**. A link to the Source Data for this figure is provided in the Data Availability statement.

Supplementary Figure 9

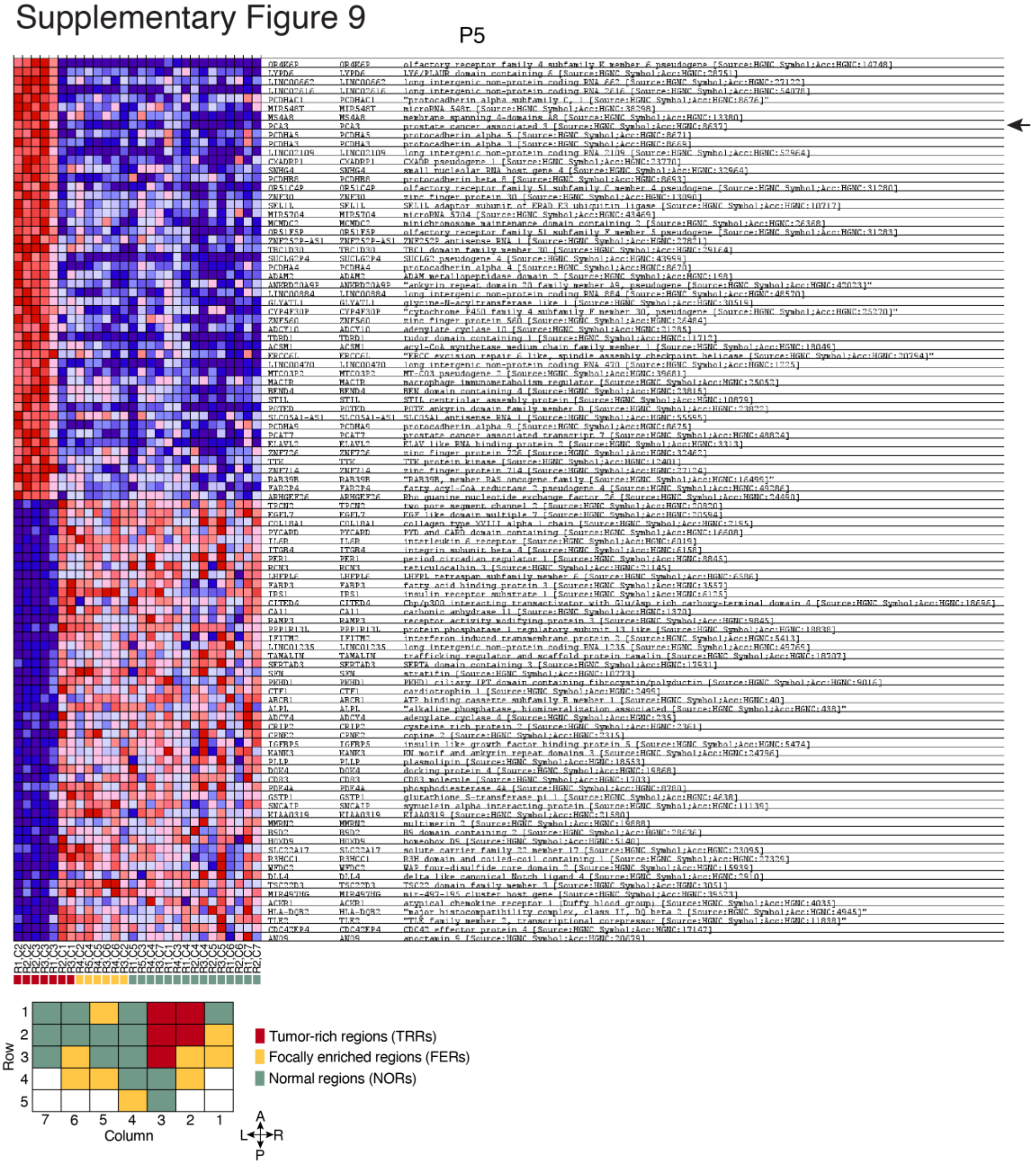

Supplementary Fig. 9. Gene set enrichment analysis (GSEA) comparing tumor-rich regions (TRRs) versus focally enriched regions (FERs) and normal regions (NORs) in prostate sample P5. The top 50 up- and down-regulated genes are shown. The arrow on the right indicates a prostate cancer biomarker gene (*PCA3*) that is upregulated in TRRs. The tissue map on the bottom left is as in **Fig. 1c**. A link to the Source Data for this figure is provided in the Data Availability statement.

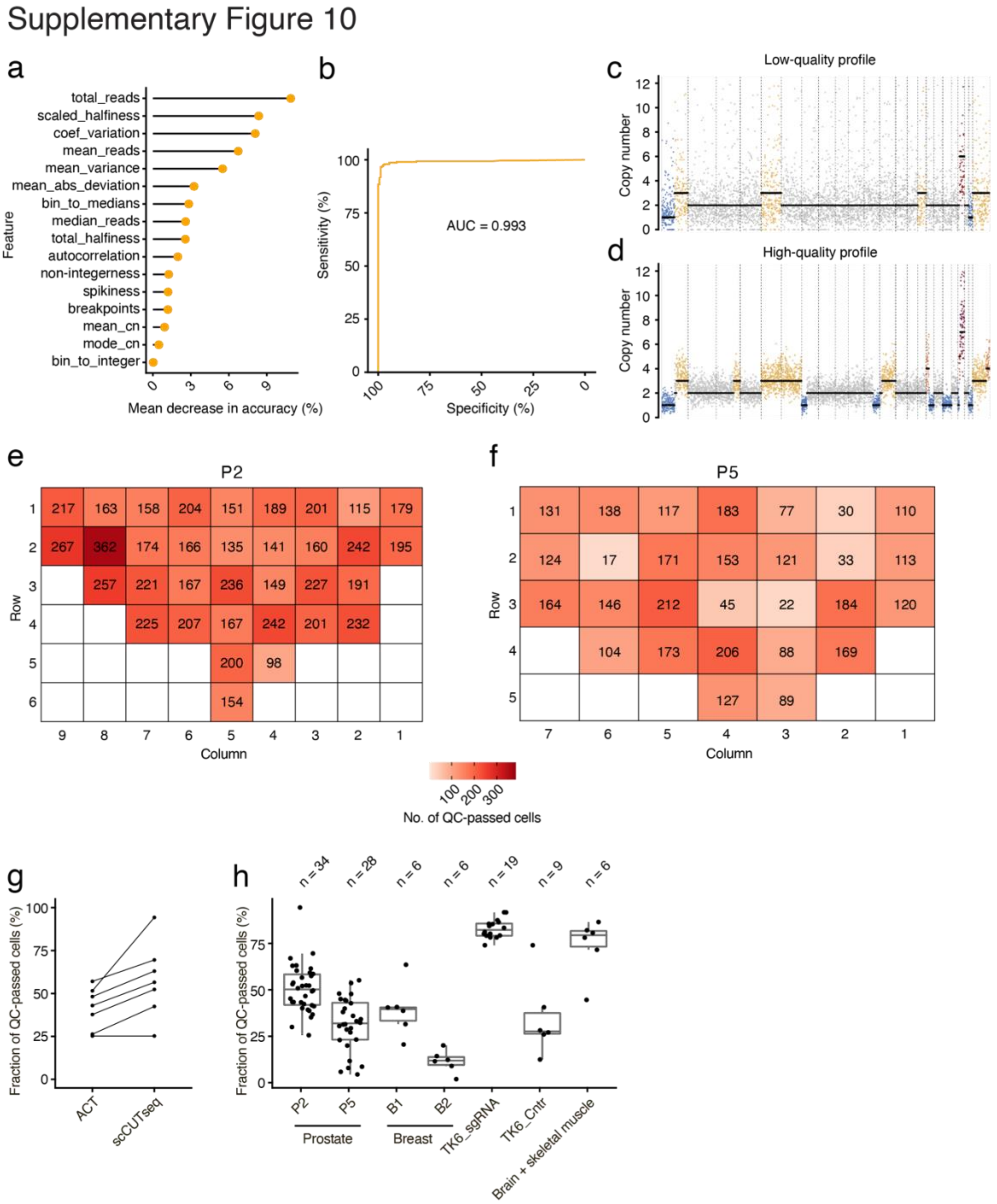

Supplementary Fig. 10. Random forest classifier of single-cell copy number profiles. (**a**) Ranked importance of the features used by the random forest classifier trained on scCUTseq data to distinguish between high- and low-quality copy number profiles. See **Supplementary Data 2** for a description of each feature. (**b**) Receiver operating characteristic curve analysis of the random forest classifier performance. AUC, area under the curve. (**c, d**) Examples of low-quality (c) and high-quality (d) quality scCUTseq copy number profiles (500 kb resolution).

Low-quality profiles are discarded by the random forest classifier, whereas high-quality profiles are retained for further analysis. Each dot represents a 500 kilobases (kb) genomic bin. Black, blue, yellow, and red dots indicate, respectively, copy number levels determined by circular binary segmentation, chromosomal deletions, gains, and amplifications. (**e, f**) Heatmaps displaying the number of cells with high-quality scCUTseq copy number profiles retained by the random forest classifier, for each region in prostate samples P2 (e) and P5 (f) profiled by scCUTseq. (**g**) Comparison of the fraction of cells with high-quality copy number profiles that passed quality control (QC) by the random forest classifier, for six regions profiled by both ACT and scCUTseq in prostate sample P2 (see **Supplementary Fig. 2c** for a map of the regions). (**h**) Distributions of the fraction of cells with high-quality copy number profiles obtained in different scCUTseq experiments performed on different cell/tissue types. Copy number profiles of brain and breast samples are shown in **Supplementary Fig. 12 and 15**, respectively. TK6_sgRNA and TK6_Cntr cells are described in **Supplementary Fig. 3**. *n*, number of sequencing libraries (see **Supplementary Data 7** for a description of each library). In all boxplots, each box spans from the 25th to the 75th percentile and whiskers extend from –1.5×IQR to +1.5×IQR from the closest quartile, where IQR is the inter-quartile range. Each dot in the boxplots represents one library (see **Supplementary Data 7** for a list of all libraries sequenced in this study). A link to the Source Data for this figure is provided in the Data Availability statement.

Supplementary Figure 11

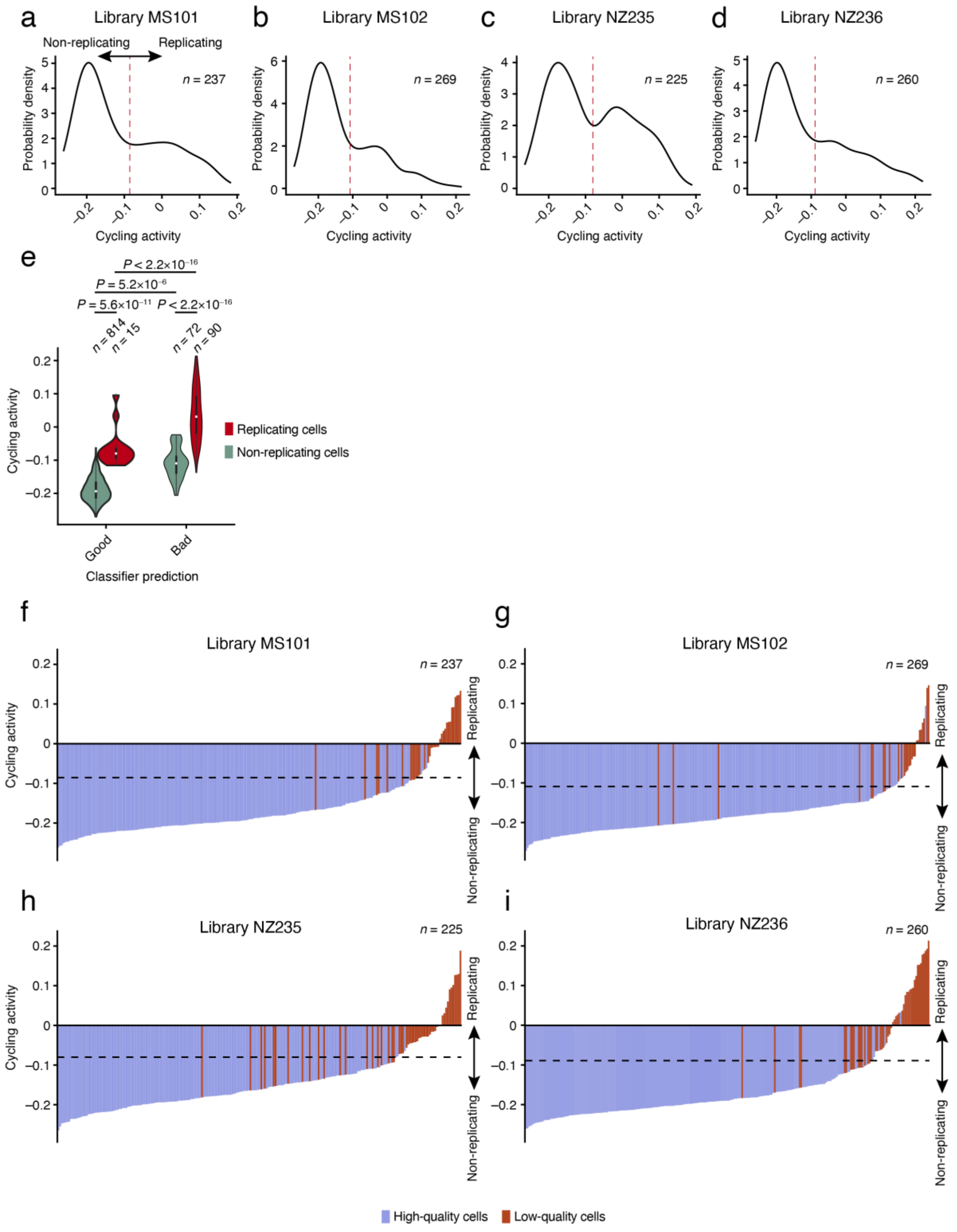

Supplementary Fig. 11. Inference of ploidy and replication status of cells profiled by scCUTseq. (**a-d**) Distributions of cycling activity scores computed by scAbsolute[1] on a total of 991 cells from four scCUTseq libraries derived from prostate sample P2 (MS101 and MS102)

and P5 (NZ235 and NZ236). The vertical dashed red lines demarcate the boundary between replicating and non-replicating cells. *n*, number of single cells analysed by scAbsolute in each library. See **Supplementary Data 7** for summary statistics of each library. (**e**) Distributions of scAbsolute cycling activity scores for the 991 single cells from the four libraries shown in (a-d) classified as 'good' or 'bad' based on their copy number profiles using the random forest classifier described in **Supplementary Fig. 10**, and as 'replicating' or 'not-replicating' based on the scAbsolute cycling activity threshold displayed in (a-d) (vertical dashed red lines). *n*, number of single cells in each group. Violin plots extend from minimum to maximum, each box in the boxplot inside each violin spans from the 25th to the 75th percentile and whiskers extend from –1.5×IQR to +1.5×IQR from the closest quartile, where IQR is the inter-quartile range. Black dots, outliers. (**f-i**) Waterfall plots of the scAbsolute cycling activity score for each cell in same four libraries shown in (a-d). Each bar represents one cell and is colored based on the quality of the corresponding copy number profile as assessed by the random forest classifier described in **Supplementary Fig. 10**. *n*, number of single cells in each library. A link to the Source Data for this figure is provided in the Data Availability statement.

Supplementary Figure 12

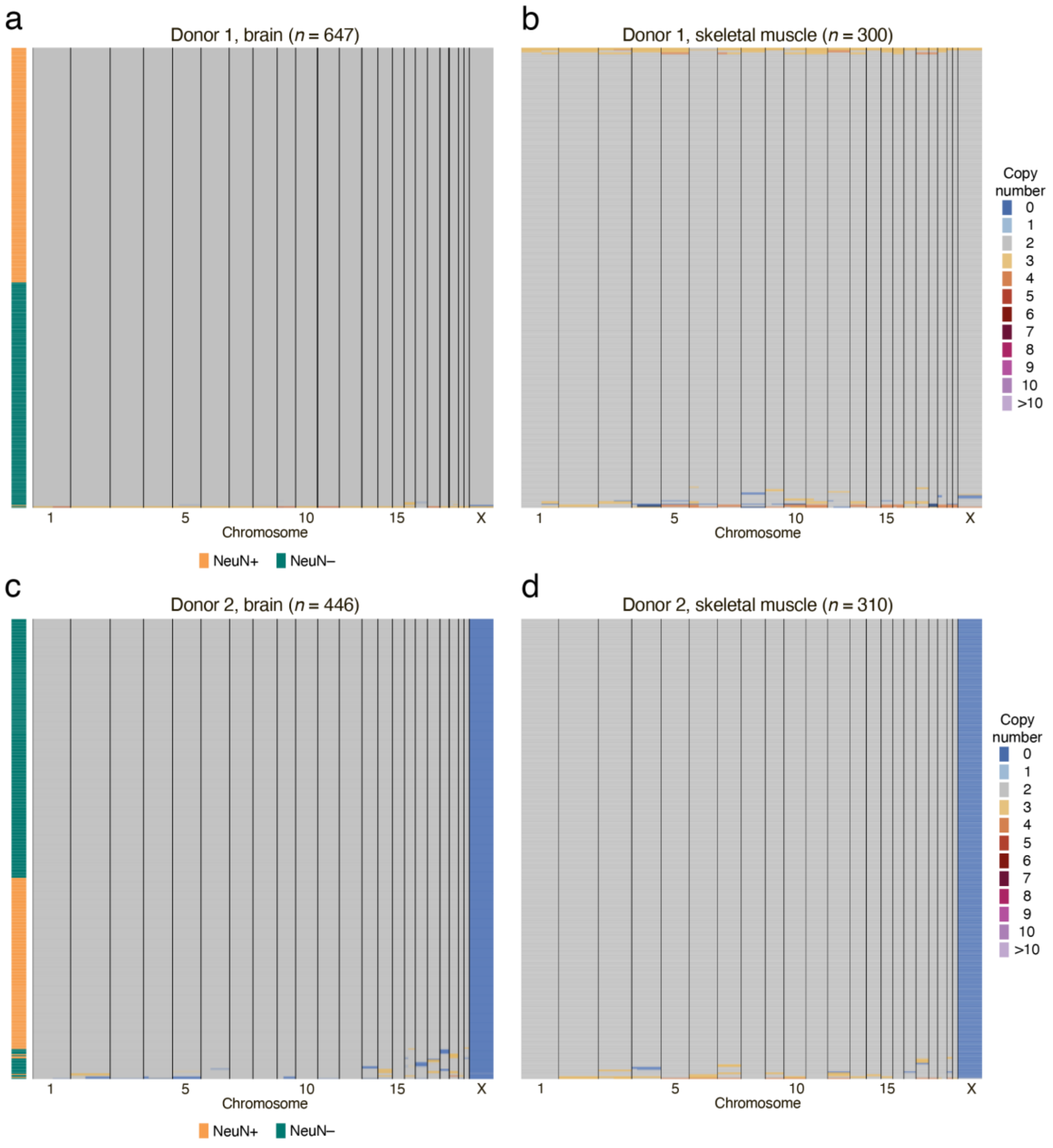

Supplementary Fig. 12. Absence of gross copy number artefacts in scCUTseq. (**a, b**) Single-cell copy number profiles (500 kb resolution) obtained by applying scCUTseq to nuclei extracted from pre-frontal cortex (brain) and skeletal muscle from one donor in the Donatum autopsy program (see **Methods and Supplementary Data 1**). Brain nuclei were sorted in neuronal (NeuN+) and non-neuronal (NeuN–) populations by FACS. *n*, number of single cells displayed. As it can be seen, the vast majority of nuclei sequenced shows a diploid copy number state, with very few cells displaying copy number alterations, indicating that the whole-genome amplification step in scCUTseq does not cause overt copy number artefacts. (**c, d**) As in (a, b)

but for a different donor. A link to the Source Data for this figure is provided in the Data Availability statement.

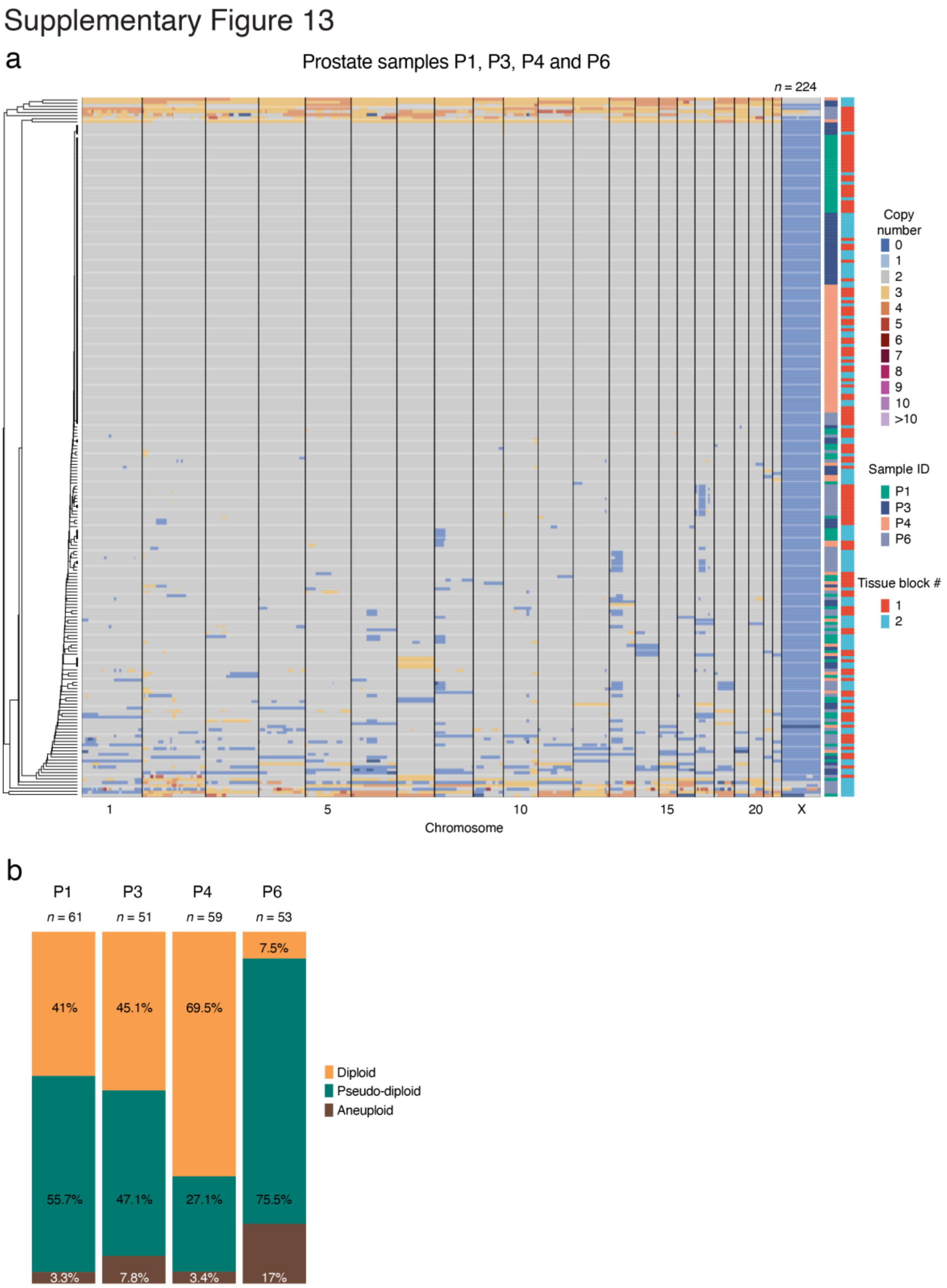

Supplementary Fig. 13. Single-cell copy number profiles (500 kb resolution) for the other four prostatectomy samples (P1, P3, P4, P6) that were profiled by scCUTseq. Two regions (tissue blocks) were profiled in each sample. (**a**) Hierarchically clustered scCUTseq profiles from all

the cells sequenced in the four samples. (**b**) Fraction of diploid, pseudo-diploid and aneuploid cells in the four samples shown in (a). *n*, number of single cells with high-quality copy number profiles analyzed. A link to the Source Data for this figure is provided in the Data Availability statement.

Supplementary Figure 14

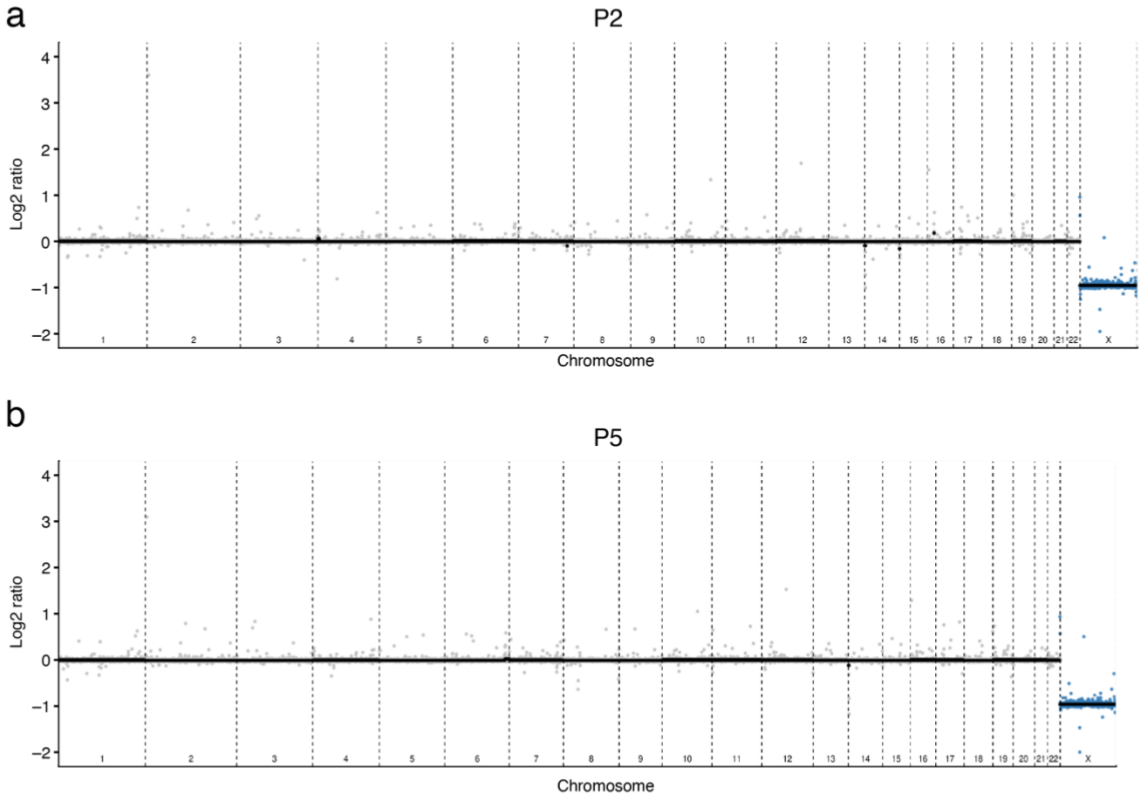

Supplementary Fig. 14. Absence of germline copy number variants in the donors of prostate samples P2 (**a**) and P5 (**b**) profiled by scCUTseq. Libraries for whole-genome sequencing (WGS) were prepared from peripheral blood gDNA using a commercial kit (NEBNext), as described in the **Supplementary Methods**. Each gray dot corresponds to a 250 kb genomic bin. A link to the Source Data for this figure is provided in the Data Availability statement.

Supplementary Figure 15

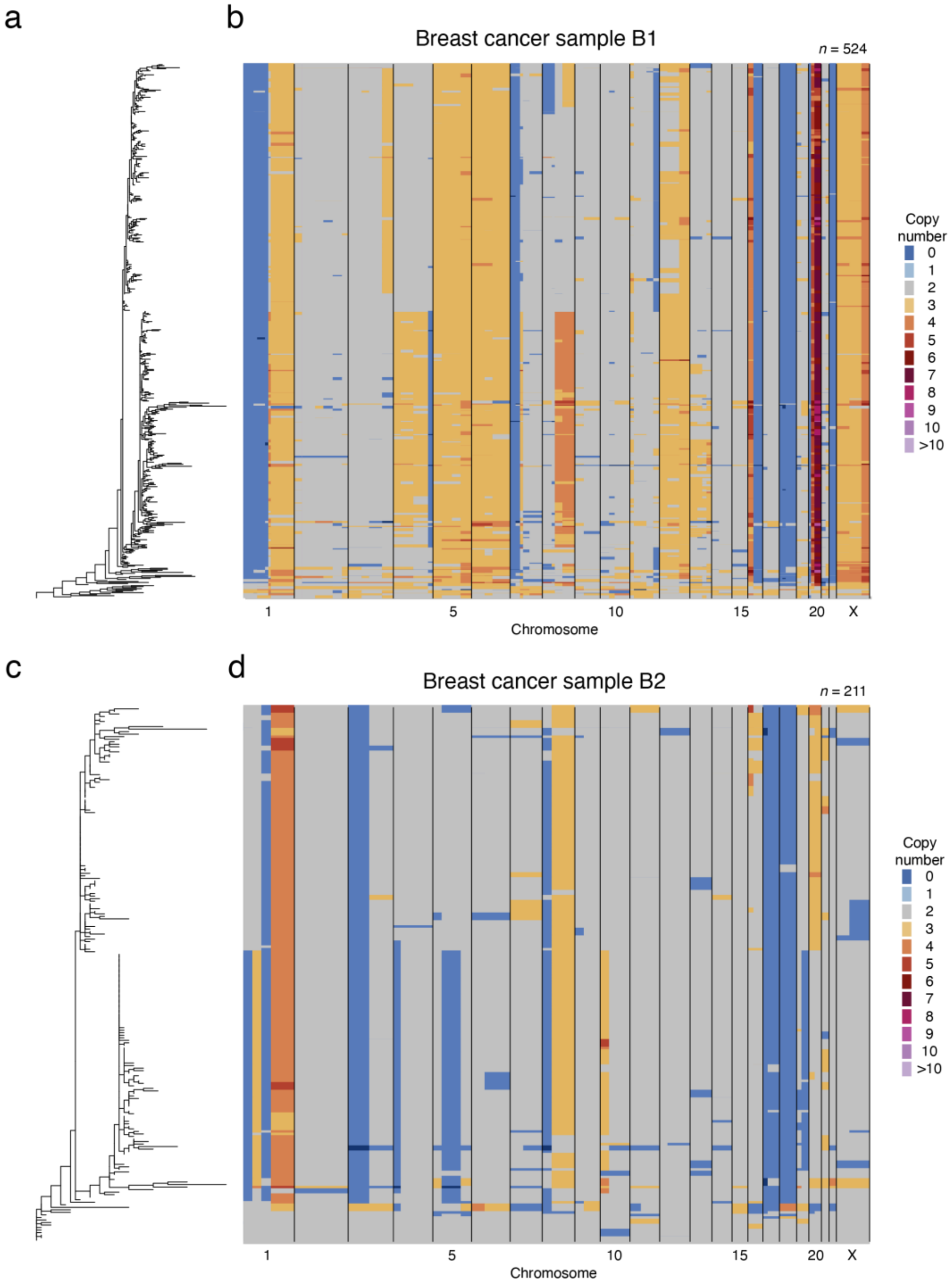

Supplementary Fig. 15. (**a**) Phylogenetic Newick tree of single cells extracted from a breast cancer surgical biopsy and profiled by scCUTseq (see **Methods**). The tree was generated by *MEDICC2*[2] (see **Methods**). Each leaf in the tree corresponds to one cell. (**b**) Copy number profiles (500 kb resolution) of the cells shown in (b). (**c, d**) As in (a, b) but for another breast

cancer specimen. A link to the Source Data for this figure is provided in the Data Availability statement.

Supplementary Figure 16

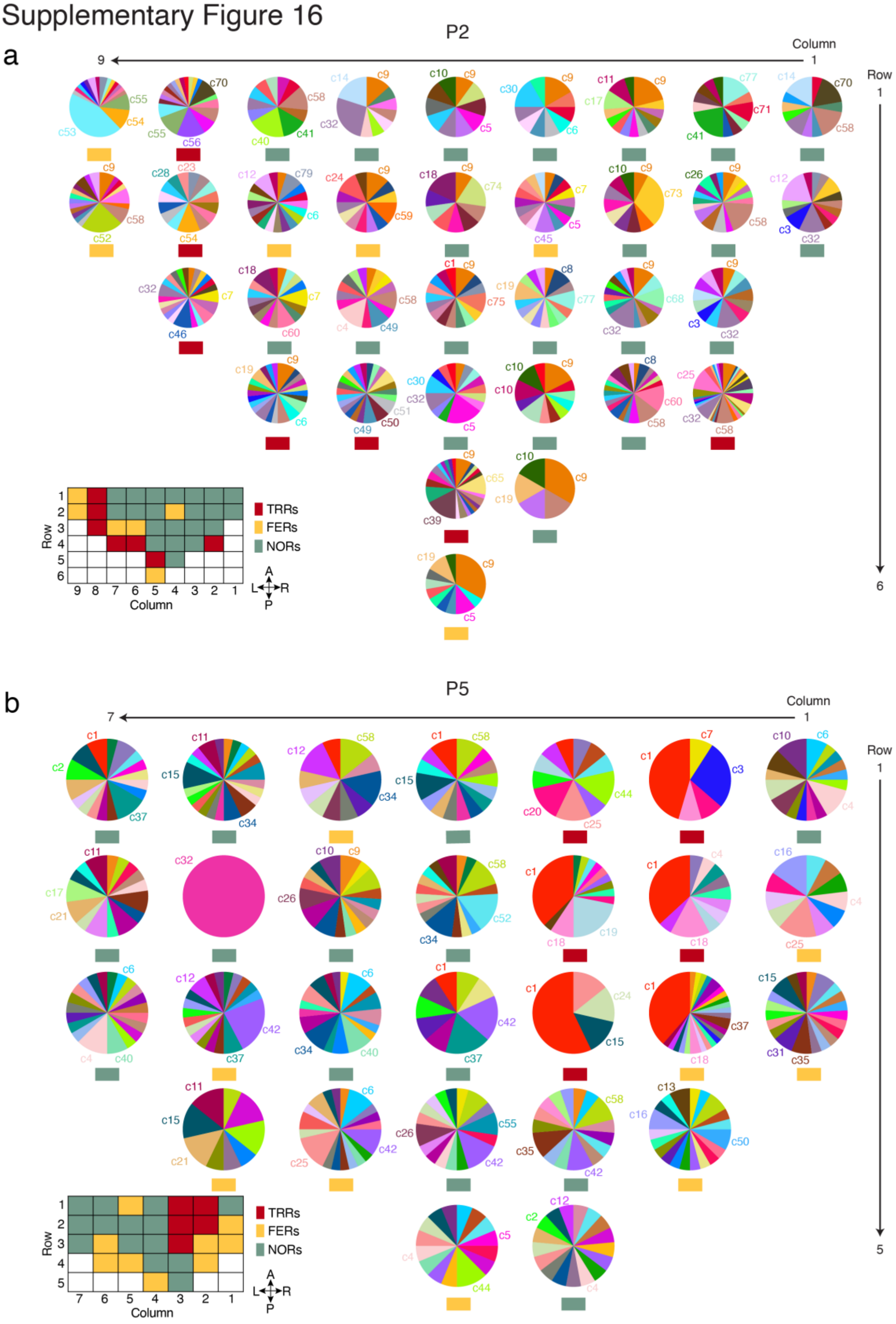

Supplementary Fig. 16. Proportion of pseudo-diploid subclones in each of the regions profiled by scCUTseq in prostate samples P2 (**a**) and P5 (**b**). Each pie chart corresponds to one tissue

cube (region). Each pie chart is color-coded (bottom rectangles) based on the pathological classification of the corresponding region as shown in the prostate midsection map on the bottom left. The anatomical orientation of the midsection is shown by the four arrows on the bottom right. A, anterior. P, posterior. L, left. R, right. TRRs, tumor-rich regions. FERs, focally enriched regions. NORs, normal regions. For each region, the three most frequent subclones (c) are indicated. Column and row numbers shown on the top and right side of the pie charts, respectively, are as those shown on the bottom and left side, respectively, of the corresponding tissue map displayed on the left. A link to the Source Data for this figure is provided in the Data Availability statement.

Supplementary Figure 17

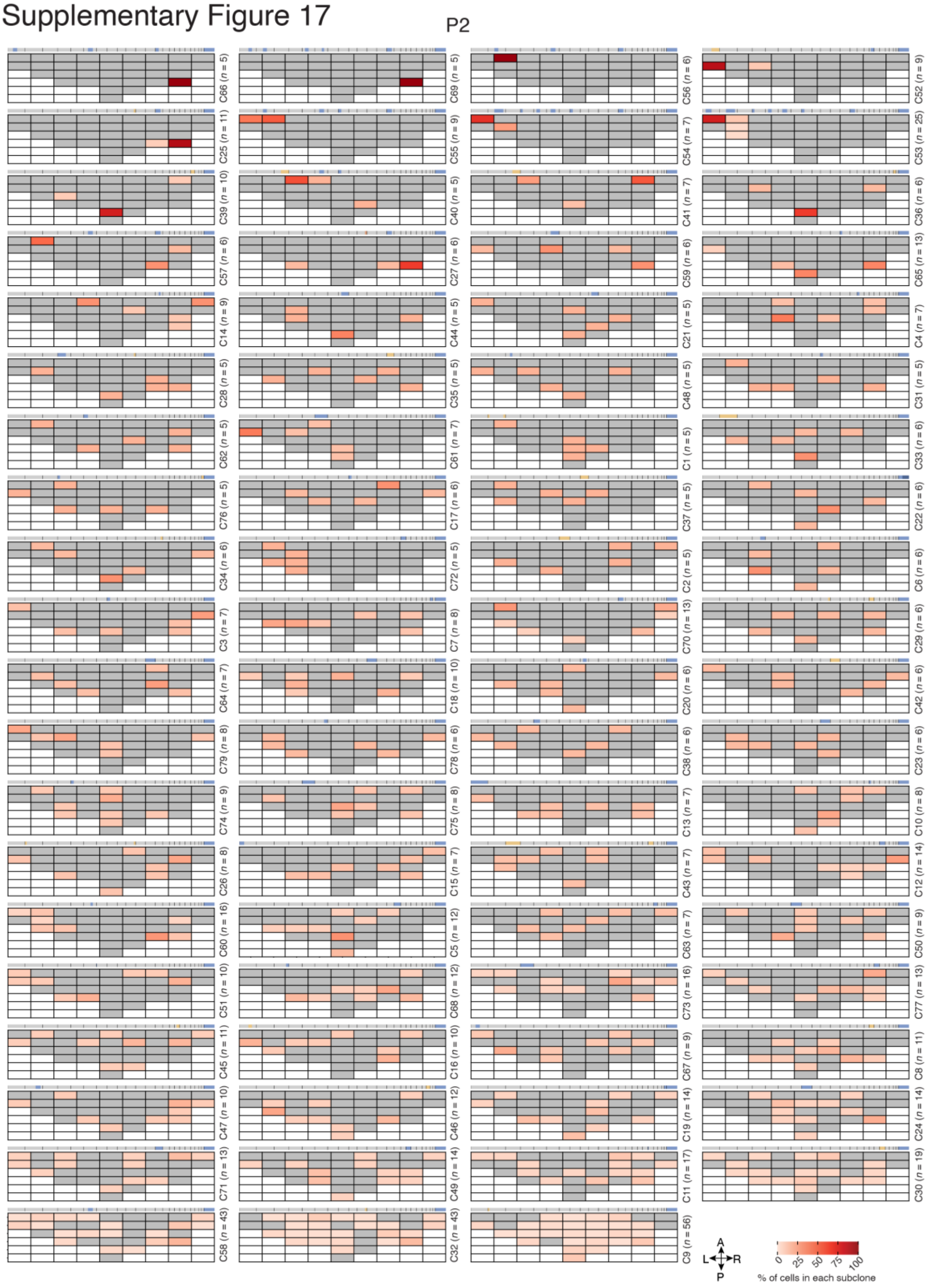

Supplementary Fig. 17. Spatial distribution of the pseudo-diploid subclones (C) identified in prostate sample P2. *n*, number of pseudo-diploid cells in each subclone. Each grid represents the tissue distribution of the corresponding subclone (indicated on the right side) and the regions

in which the subclone was found are color-coded based on the percentage of cells belonging to that subclone. Grey cells indicate tissue regions in which the corresponding subclone was not detected. White cells indicate absence of prostate tissue. The bar above each grid shows the genome-wide median copy number profile of the corresponding subclone. Blue bars indicate deletions, orange bars amplifications. Vertical black bars mark the boundaries between consecutive chromosomes. The anatomical orientation of the grids is shown by the four arrows on the bottom right. A, anterior. P, posterior. L, left. R, right. A link to the Source Data for this figure is provided in the Data Availability statement.

Supplementary Figure 18

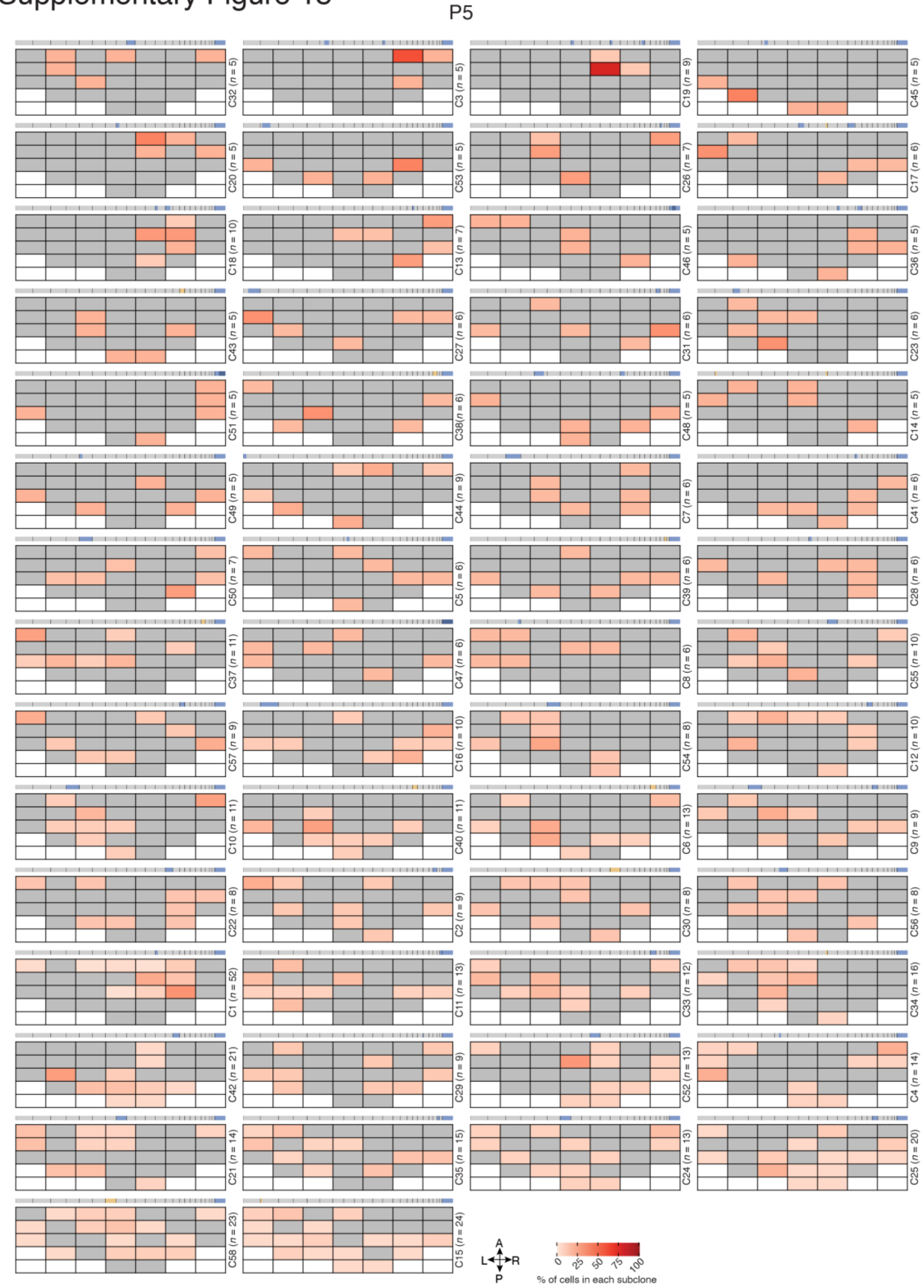

Supplementary Fig. 18. Spatial distribution of the pseudo-diploid subclones (C) identified in prostate sample P5. *n*, number of pseudo-diploid cells in each subclone. Each grid represents the tissue distribution of the corresponding subclone (indicated on the right side) and the regions

in which the subclone was found are color-coded based on the percentage of cells belonging to that subclone. Grey cells indicate tissue regions in which the corresponding subclone was not detected. White cells indicate absence of prostate tissue. The bar above each grid shows the genome-wide median copy number profile of the corresponding subclone. Blue bars indicate deletions, orange bars amplifications. Vertical black bars mark the boundaries between consecutive chromosomes. The anatomical orientation of the grids is shown by the four arrows on the bottom right. A, anterior. P, posterior. L, left. R, right. A link to the Source Data for this figure is provided in the Data Availability statement.

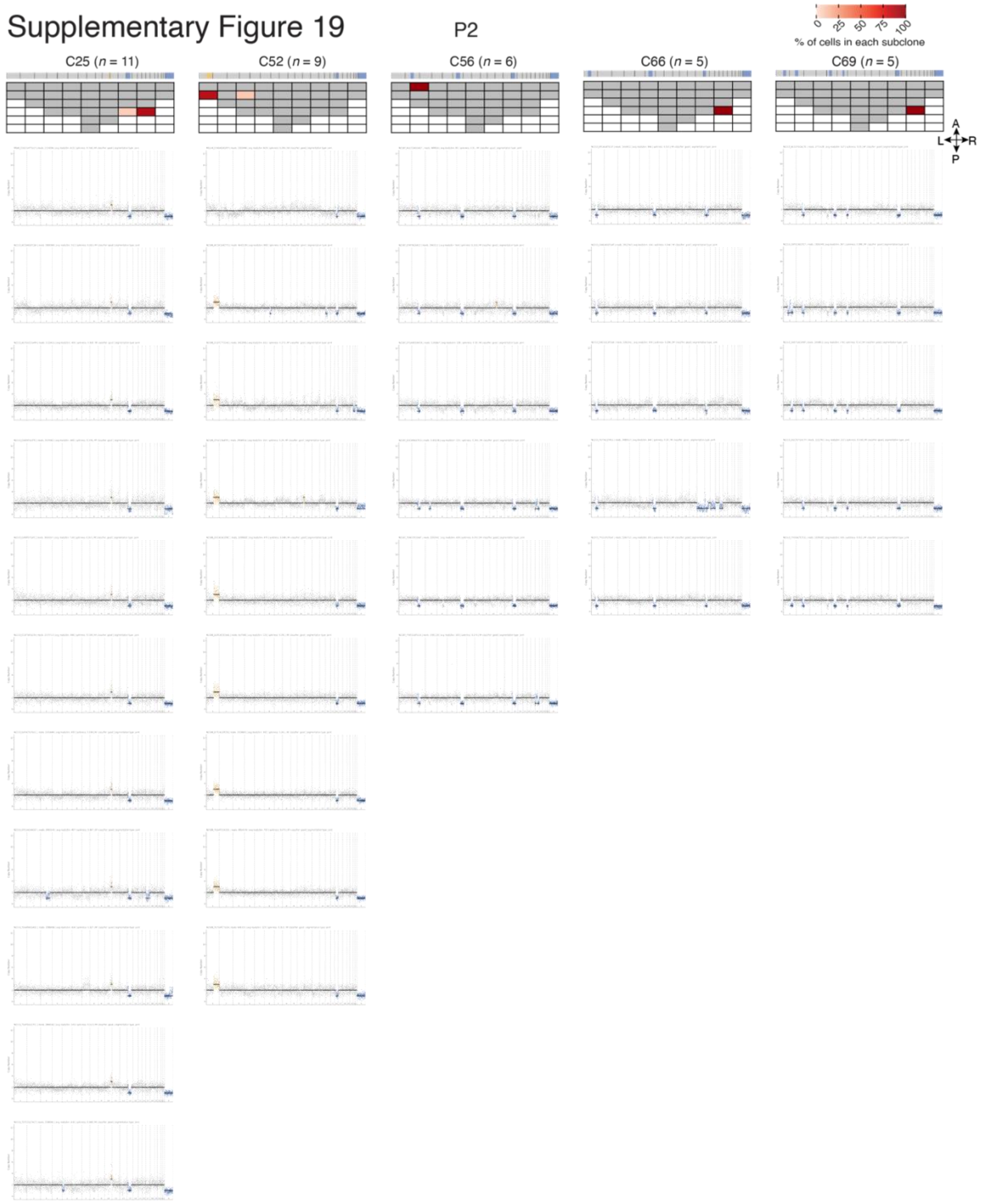

Supplementary Fig. 19. Individual copy number profiles (500 kb resolution) of the pseudo-diploid cells in each of the five most localized subclones (C) identified in prostate sample P2. *n*, number of pseudo-diploid cells in each subclone. In each copy number plot, dots represent 500 kb genomic bins. Orange and blue dots indicate, respectively, amplifications and deletions. Grey dots indicate bins belonging to normal (diploid) genomic segments. The grids on the top represent the tissue distribution of the corresponding subclone. Grey cells indicate tissue

regions in which the corresponding subclone was not detected. White cells indicate absence of prostate tissue. The bar above each grid shows the genome-wide median copy number profile of the corresponding subclone. The anatomical orientation of the grids is shown by the four arrows on the right. A, anterior. P, posterior. L, left. R, right. A link to the Source Data for this figure is provided in the Data Availability statement.

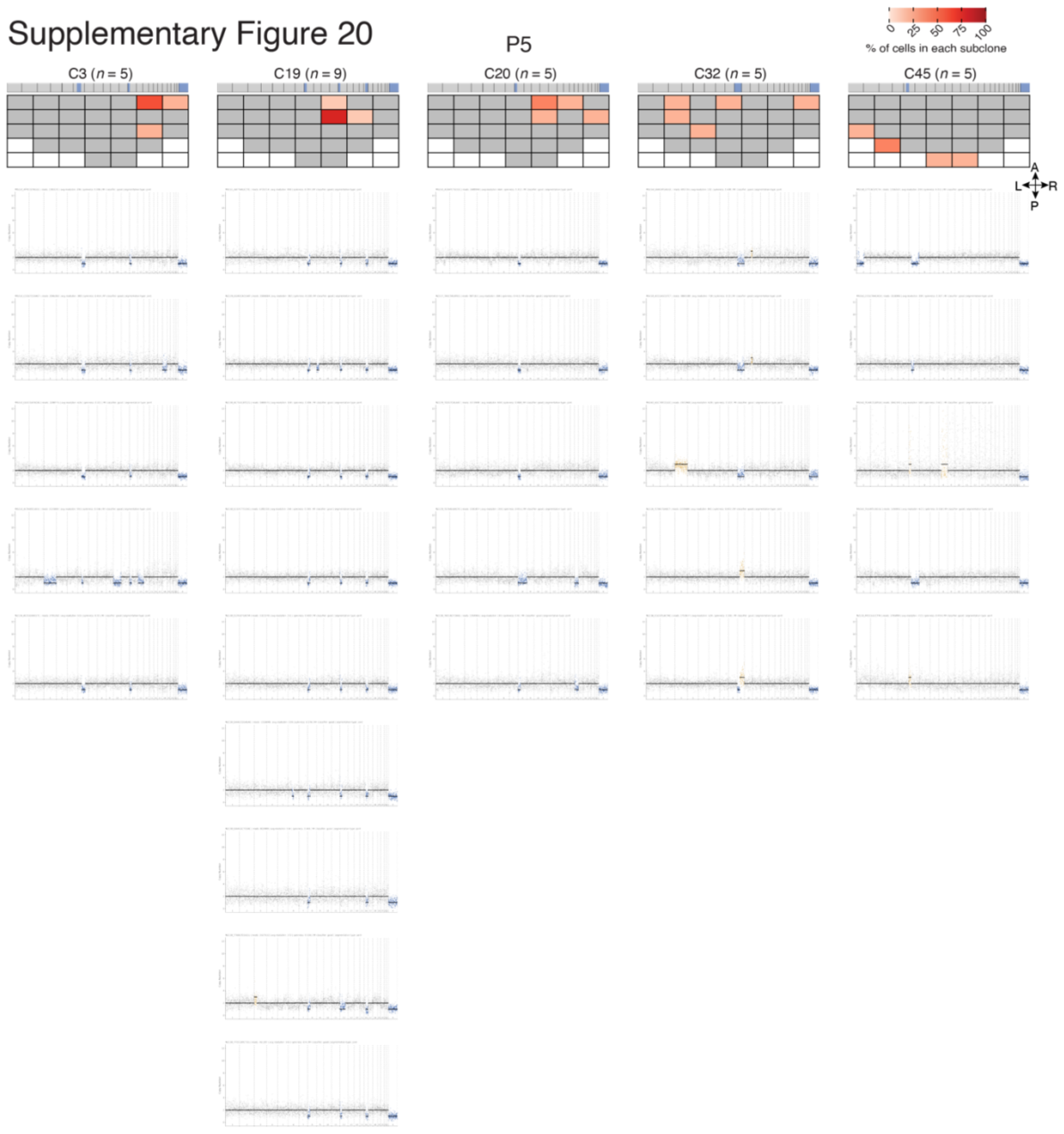

Supplementary Fig. 20. Individual copy number profiles (500 kb resolution) of the pseudo-diploid cells in each of the five most localized subclones (C) identified in prostate sample P5. *n*, number of pseudo-diploid cells in each subclone. In each copy number plot, grey dots represent 500 kb genomic bins. Orange and blue dots indicate, respectively, amplifications and deletions. Grey dots indicate bins belonging to normal (diploid) genomic segments. The grids on the top represent the tissue distribution of the corresponding subclone. Grey cells indicate tissue regions in which the corresponding subclone was not detected. White cells indicate absence of prostate tissue. The bar above each grid shows the genome-wide median copy number profile of the corresponding subclone. The anatomical orientation of the grids is shown

by the four arrows on the right. A, anterior. P, posterior. L, left. R, right. A link to the Source Data for this figure is provided in the Data Availability statement.

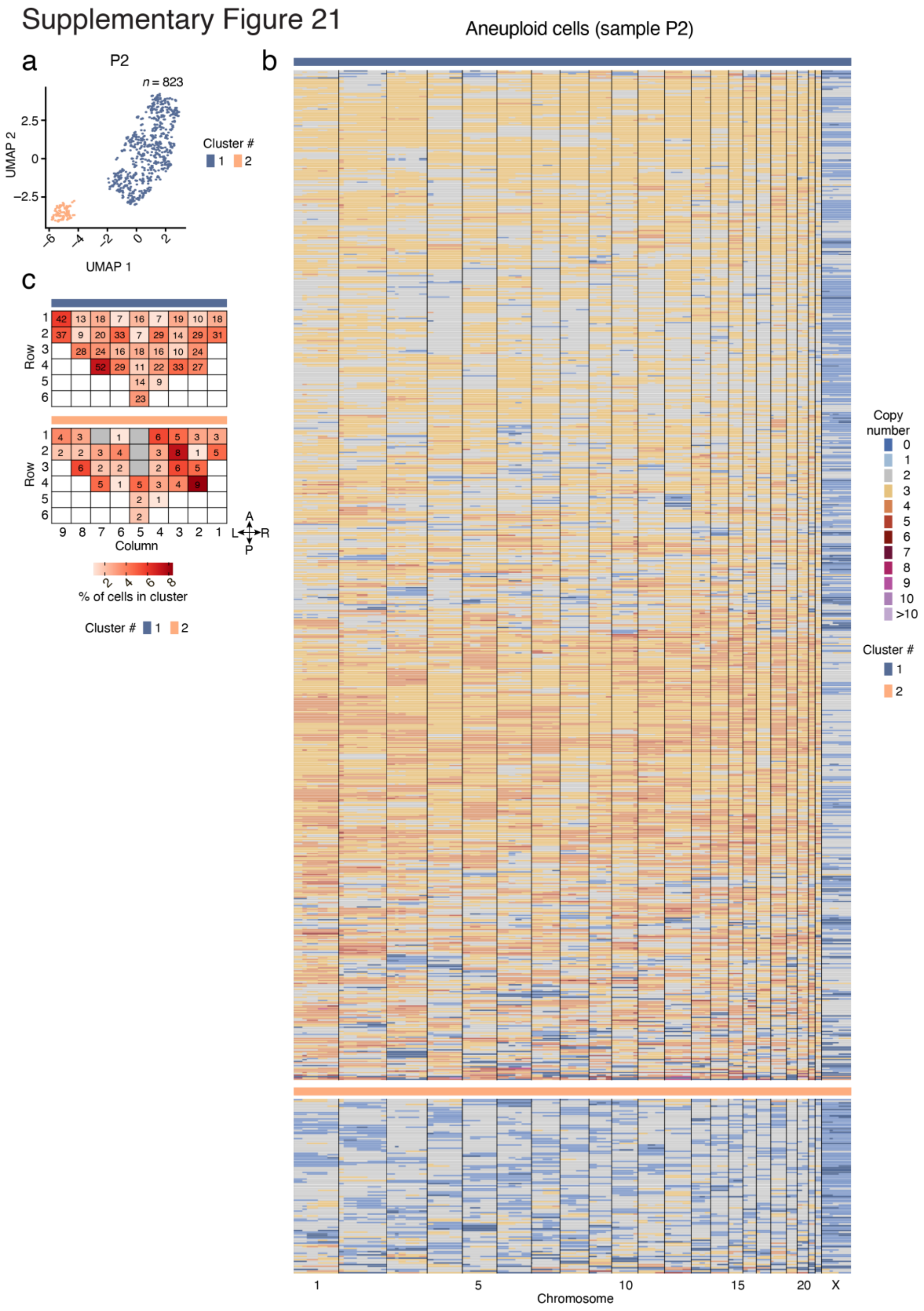

Supplementary Fig. 21. Characteristics and spatial distribution of aneuploid cells in prostate sample P2. (**a**) Uniform Manifold Approximation and Projection (UMAP) dimensionality

reduction of aneuploid cell SCNA profiles. *n*, number of cells. Dots of the same color belong to the same cluster. Each dot represents a single cell. (**b**) Single-cell copy number profiles of aneuploid cells in each UMAP cluster shown in (a). (**c**) Maps showing the tissue distribution of aneuploid cells for each UMAP cluster in (a). In each grid, each cell represents a tissue region from which nuclei were isolated and profiled by scCUTseq. The numbers in each cell of the grids indicate the number of aneuploid cells assigned to the indicated cluster in that region. Grey cells mark tissue regions in which no aneuploid cell was detected. White cells indicate absence of prostate tissue. The anatomical orientation of the maps is shown by the four arrows on the bottom right. A, anterior. P, posterior. L, left. R, right. A link to the Source Data for this figure is provided in the Data Availability statement.

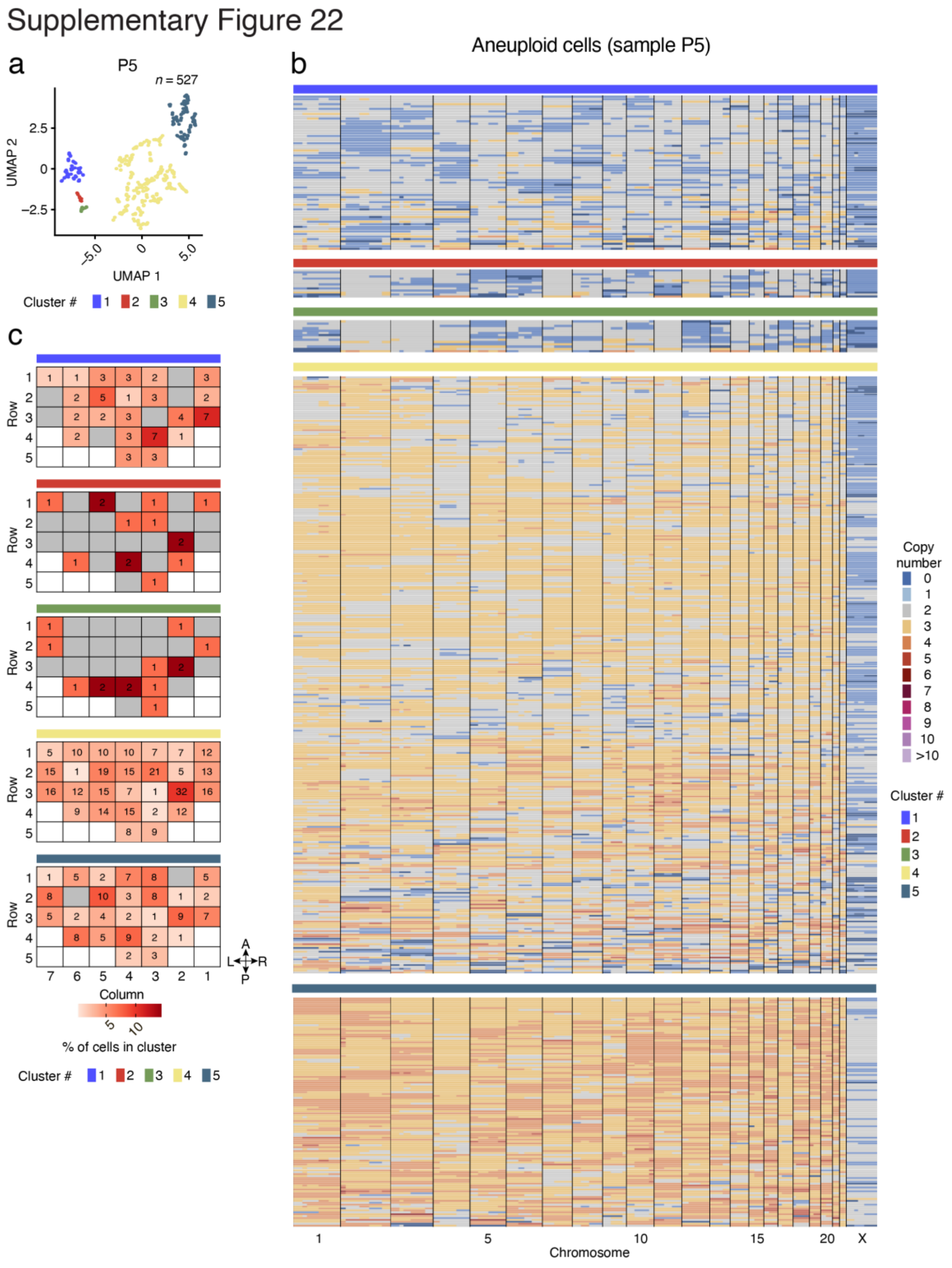

Supplementary Fig. 22. Characteristics and spatial distribution of aneuploid cells in prostate sample P5. (**a**) Uniform Manifold Approximation and Projection (UMAP) dimensionality reduction of aneuploid cell SCNA profiles. *n*, number of cells. Dots of the same color belong to the same cluster. Each dot represents a single cell. (**b**) Single-cell copy number profiles of

aneuploid cells in each UMAP cluster shown in (a). (**c**) Maps showing the tissue distribution of aneuploid cells for each UMAP cluster in (a). In each grid, each cell represents a tissue region from which nuclei were isolated and profiled by scCUTseq. The numbers in each cell of the grids indicate the number of aneuploid cells assigned to the indicated cluster in that region. Grey cells mark tissue regions in which no aneuploid cell was detected. White cells indicate absence of prostate tissue. The anatomical orientation of the maps is shown by the four arrows on the bottom right. A, anterior. P, posterior. L, left. R, right. A link to the Source Data for this figure is provided in the Data Availability statement.

Supplementary Figure 23

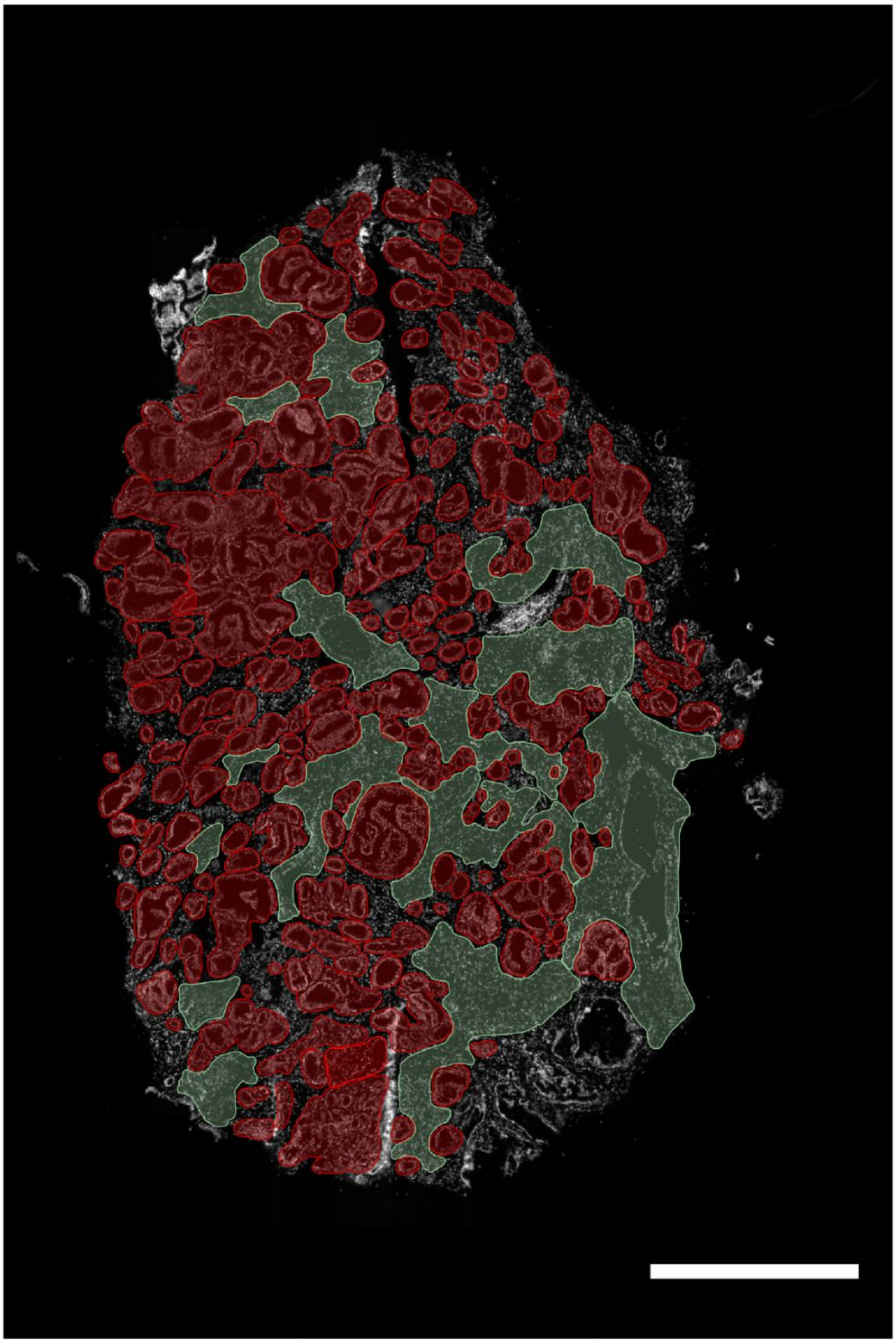

Supplementary Fig. 23. QuPath annotation of a tissue section adjacent to the one profiled by DNA FISH in prostate sample P6 (see **Fig. 3**). Red, neoplastic prostate glands. Green, stroma. Scale bar, 1 mm.

Supplementary Figure 24

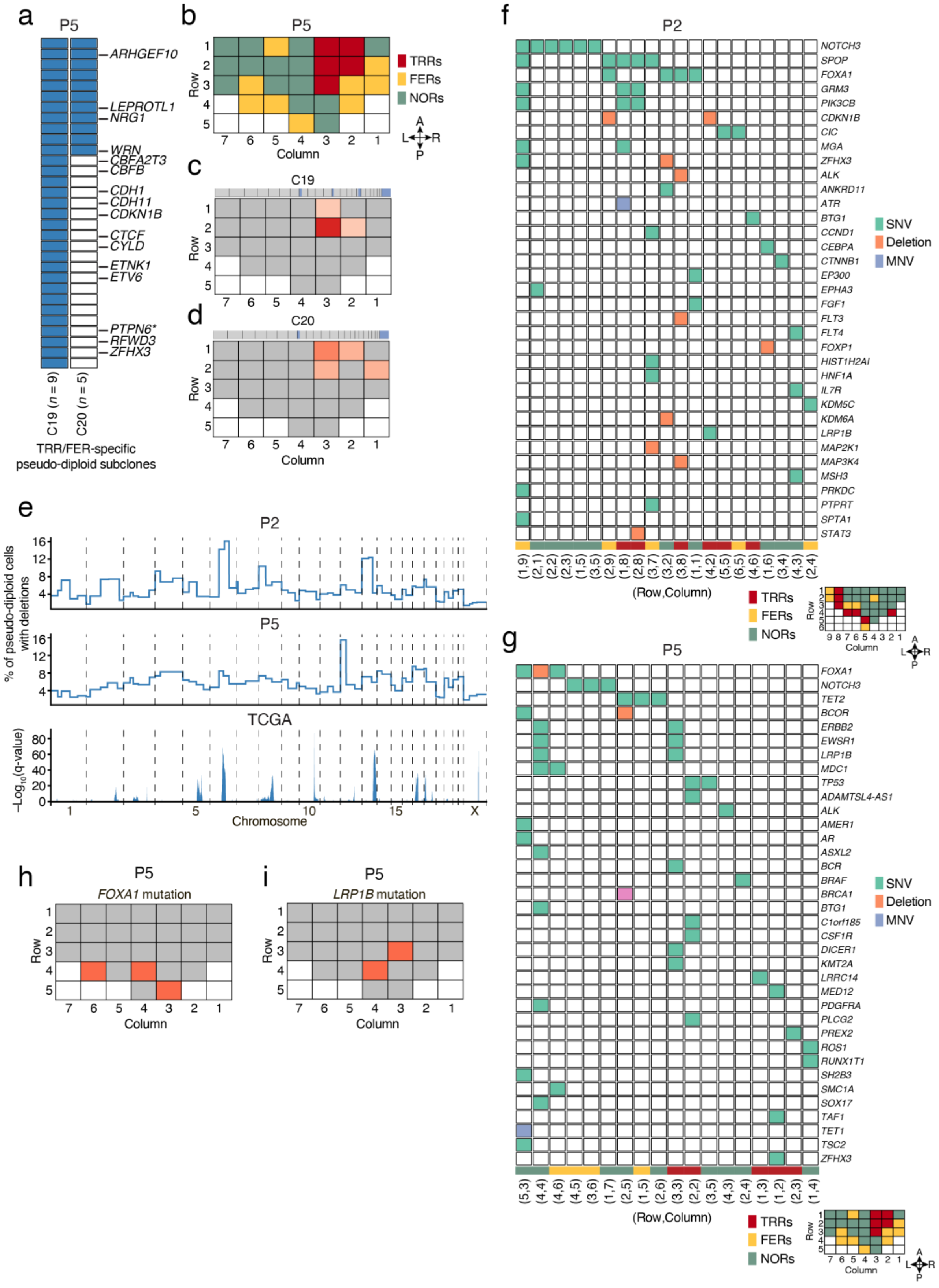

Supplementary Fig. 24. Loss of tumor-suppressor genes and mutations in prostate cancer associated genes in prostate sample P5. (**a**) OncoPrint plot showing genes classified as tumor-

suppressor genes (TSGs) in the Catalogue of Somatic Mutations in Cancer (COSMIC) that were deleted (blue rectangles) in two pseudo-diploid subclones (C) localized exclusively in tumor-rich regions (TRRs) or focally enriched regions (FERs) in prostate sample P5. *n*, number of pseudo-diploid cells in each subclone. Asterisks indicate genes annotated in COSMIC both as TSGs and as oncogenes (context-dependent TSGs). (**b**) As in **Fig. 1d**. (**c, d**) Spatial distribution of the two pseudo-diploid subclones (C) shown in (a). The median copy number profile of each subclone is shown on top of the corresponding tissue map. (**e**) Genome-wide deletion frequency in the pseudo-diploid cells identified in prostate samples P2 (top) and P5 (middle) compared to TCGA-PRADs (bottom). (**f**) OncoPrint plot showing the COSMIC genes mutated and type of mutation in each region in prostate sample P2. SNV, single-nucleotide variant (green rectangles). MNV, multi-nucleotide variant. The tissue map on the bottom right is as the one shown in **Fig. 1c**. (**g**) As in (e) but for prostate sample P5. The tissue map on the bottom right is as the one shown in **Fig. 1d**. (**h, i**) Schematic maps showing where the indicated genes were found mutated in sample P5. Grey cells indicate tissue regions in which the indicated gene was not altered. White cells indicate absence of tissue. The anatomical orientation of the maps in (c, d) and (h, i) is as in (b). A link to the Source Data for this figure is provided in the Data Availability statement.

2. Supplementary Methods

RNA-seq

We prepared RNA-seq libraries from the total RNA extracted from each region in the P3 and P6 prostate samples (see **Supplementary Table 1**) using the TruSeq Stranded Total RNA Library Prep kit (Illumina, cat. no. 20020597) with an RNA input ranging from 800 ng to 1 µg of total RNA with RIN > 6. After removing rRNAs, we fragmented the remaining RNAs for 5 min, followed by cDNA synthesis, end-repair, 3'-adenylation, and adapter ligation following the manufacturer's instructions. Lastly, we sequenced pooled libraries on Illumina NovaSeq 6000 with a loading concentration equal to 1.2 nM and paired-end 100 bp sequencing mode.

To analyze the data, we aligned paired-end reads to the GRCh38 genome assembly using the *HISAT2* aligner[3]. We filtered the resulting bam files for ribosomal RNA sequences using the *RSeQC* module *split_bam.by*. We estimated gene expression abundance using the *FeatureCounts* function in the *Rsubread* (version 2.14.2)[4] R package and GENCODE (v40) genomic features annotation. We then subjected gene expression counts to filtering of lowly expressed features, trimmed-mean of M-values (TMM) normalization, and counts per million (CPM) calculation using the *edgeR* (version 3.42.4)[5] R package. For class discovery, we used a consensus based non-negative matrix factorization (NMF) approach implemented in the *NMF* (version 0.26)[6] R package. We carried out unsupervised count matrix decomposition on the top 25% most variable features for increasing numbers of factorization rank, ranging from 2 to 6, and selecting 200 iterations. Lastly, we used the Gene Set Enrichment Analysis (GSEA) software (version 4.2.3)[7] and human *MSigDB c2.all.v2023.1.Hs.symbols.gmt* gene set to extract biomarkers for each supervised cluster (i.e., TRRs, FERs, and NORs).

Standard MALBAC

We washed the harvested cells in 1× PBS/5 mM EDTA at room temperature and resuspended them in the same buffer at a density of 10^6/mL. We then placed the cells either on ice for immediate sorting (live cells) or fixed them to store them for longer periods before sorting. For fixation, we added an equal volume of 1× PBS/5 mM EDTA/8% methanol-free paraformaldehyde (PFA, Thermo Fisher Scientific, cat. no. 28908) to the cell suspension and pipetted the solution up and down ten times. After 10 min incubation in darkness, we added 2.5 M glycine to each tube to reach a final concentration of 125 mM to quench any residual unreacted PFA. We then washed the cells in 1× PBS/5 mM EDTA at room temperature,

resuspended them at a density of 10^6/mL in the same buffer, and stored them at +4 °C in 1× PBS/5 mM EDTA/0.05% NaN_3. We have successfully used fixed cells prepared in this manner, that were kept at +4 °C for several weeks up to three months. Before sorting, we transferred the cells into FACS-compatible tubes, stained them with 2.46 ng/mL Hoechst 33342 (Thermo Fisher Scientific, cat. no. 62249) and incubated for 40 min at 37 °C in darkness, while rotating. We sorted fixed and live single cells in a 96-well plate pre-filled with MALBAC lysis reaction mix (5 μL of lysis buffer + 0.5 μL of lysis enzyme) using a BD FACSJazz Cell Sorter (BD Biosciences) based on forward and side scatter properties. We centrifuged the plate at 600×g for 3 min. In parallel we used one well as negative control by adding 1 μL of Nuclease-Free Water (Thermo Fisher Scientific, cat. no. 4387936) into the lysis mix and another well as positive control by adding 1 μL of gDNA extracted from fixed cells at a final concentration of 30 pg/μL into the lysis mix. We incubated the plate at 50 °C for 1 h and 80 °C for 10 min in a PCR thermocycler. After lysis, we added 31 μL of MALBAC pre- amplification reaction mix (30 μL of pre-amp buffer + 1 μL of pre-amp enzyme mix) into each well, and centrifuged the plate at 600×g for 3 min followed by incubation at 94 °C for 3 min to denature DNA and then by 8 cycles of quasilinear amplification (20 °C for 40 s, 30 °C for 40 s, 40 °C for 30 s, 50 °C for 30 s, 60 °C for 30 s, 70 °C for 4 min, 95 °C for 20 s and 58 °C for 10 s) in a PCR thermocycler. After pre-amplification, we added 30.8 μL of MALBAC amplification mix (30 μL of amp buffer + 0.8 μL of amp enzyme mix) into each well, centrifuged the plate at 600×g for 3 min followed by incubation at 94 °C for 30 s to denature DNA and then by 14 cycles of exponential amplification (94 °C for 20 s, 58 °C for 30 s, 72 °C for 3 min) in a PCR thermocycler. We performed lysis, pre-amplification and amplification steps using reagents included in the MALBAC kit (Yikon Genomics, cat. no. Y001A). We purified the amplified material from each well separately using 1.8 vol./vol. ratio of Agencourt Ampure XP beads (Beckman Coulter, Cat. No. A63881) and measured the concentration using Qubit DNA HS kit (Thermo Fisher Scientific, Cat. No. Q32851). We spared 200 ng of DNA for the following steps. First, we sheared the samples using Covaris ME220 Focused-ultrasonicator with a target peak set at 200 base pairs (bp) and then performed library preparation using the NEBNext Ultra II DNA Library Prep Kit for Illumina (NEB, cat. no. E7645S). We checked the fragment distribution of the final libraries on a Bioanalyzer 2100 using DNA HS chip (Agilent, cat. no. 5067-4626). To reduce the MALBAC reagent volumes and therefore the cost per cell, we tested different scaled-down versions of MALBAC (sMALBAC) by reducing the reagent volumes in the lysis, pre-amplification, and amplification steps 50, 100, 200 or 500 times. To this end, we first sorted fixed or live single cells in a 384-well plate pre-filled with 5 μL of Vapor-Lock

(Qiagen, cat. no. 981611) and dispensed the scaled reagent volumes using the I.DOT nanodispenser (CELLINK) in 384-well plates. We then collected the MALBAC products manually from each well by adding 5 µL of Nuclease-Free Water to each well, and then fragmented each sample, and prepared sequencing library as described above.

Assessment of scCUTseq sensitivity

Generation of Cas9-expressing TK6 cells. We cultured TK6 lymphoblastoid cells in RPMI-1640 medium with 5 % horse serum (Gibco, cat. no. 11510516), supplemented with 2 mM L-glutamine (Gibco, cat. no. A2916801), 100 U/ml penicillin/100 µg/ml streptomycin (Gibco, cat. no. 15140122), and 100 µM sodium-pyruvate (Gibco, cat. no. 11360070) at 37 °C and 5% CO2. To establish TK6-Cas9 cells stably expressing SpCas9, we transduced the parental cells with viral particles produced using a Cas9 lentiviral expression vector (pLenti-Cas9 Blast, Addgene cat. no. 52962, kind gift from Feng Zhang). Briefly, we produced lentiviral particles in HEK293T cells by transfecting them with 4 µg of the Cas9 lentiviral expression vector and 1 µg each of lentiviral packaging plasmids, pMDLg/pRRE, pRSV-REV, pMD2.G (Addgene, cat. no. 12251, 12253, and 12259, respectively, all kind gifts from Didier Trono) using the X-tremeGENE HP DNA Transfection Reagent (Roche, cat. no. 6366244001). We pooled the virus-containing supernatant on two consecutive days from two 10 cm dishes of HEK293 transfected with the same plasmids and concentrated the supernatant 100 times using Lenti-X Concentrator (TaKaRa, cat. no. 631231). We then added the concentrated virus to 106 TK6 cells together with 8 µg/ml of Polybrene (Sigma-Aldrich, cat. no. TR-1003-G). One day after infection, we exchanged the medium and added 5 µg/ml Blasticidin to select the cells that had been successfully transfected. We prepared single-cell clones from limiting dilutions in 96-well plates and checked each clone for Cas9 expression and activity by immunoblotting and immunofluorescence microscopy using an anti-Cas9 antibody (Active Motif, cat. no. 61577), and by performing a T7 endonuclease assay following electroporation of the cells with specific gRNAs.

Deletion induction by CRISPR/Cas9. To assess the sensitivity of scCUTseq, we generated a 7 Mb deletion on chr11 in the Cas9-expressing TK6 cell line described above. We targeted the *KMT2A* (hg38 chr11:118488514-118488533) and *HYLS1* (hg38 chr11:125899369-125899388) loci, which are approximately 7 Mb apart on the q-arm of chr11. To this end, we purchased guide RNAs (gRNAs) (*KMT2A*: TTTGGGTTTTAGTAGTCCAC; *HYLS1*: ATGGAAGAACTTCTACCTGA) from Dharmacon and assembled them into active complexes by adding crRNA-tracrRNA components (Dharmacon, cat. no. U-002005-5)

according to the manufacturer's instructions. We electroporated the sgRNA complexes to TK6-Cas9 cells established as described above, using the Neon Transfection System (Thermo Fisher Scientific, cat. no. MPK10025) with the following program: 1350 V, 10 msec, 3 pulses. We used 106 cells with a final amount of 1.6 nmol of gRNA in each electroporation. Afterwards, we kept the cells in growing medium for two days at 37 °C and 5% CO2 to allow for the 7 Mb genomic region between the *KMT2A* and *HYLS1* loci to be deleted in a fraction of the cells.

High-throughput DNA FISH. To assess the frequency of the induced 7 Mb deletion, we prepared FISH probes targeting the KMT2A-5′ (Thermo Fisher, cat. no. CTD2159M9) KMT2A-3′ (BACPAC Resources CHORI, cat. no. RP11-59N1), HYLS1-5′ (BACPAC Resources CHORI, cat. no. RP11-712D22) and HYLS-3′ (BACPAC Resources CHORI, cat. no. RP11-50B3) loci using bacterial artificial chromosomes (BACs) and performing nick translation with the Nick Translation kit (Abbott Molecular, cat. no. 7J0001) and using the following fluorescently labeled dUTPS: AlexaFluor 488-5-dUTP (Thermo Fisher Scientific, cat. no. C11397); AlexaFluor 568-5-dUTP (Thermo Fisher Scientific, cat. no. C11399); AlexaFluor 647-AHA-dUTP (Thermo Fisher Scientific, cat. no. A32763); CF405S-dUTP (Biotium, cat. no. 40004). We plated TK6-Cas9 cells onto poly-L-lysine coated 96-well glass-bottom imaging plates (PerkinElmer, cat. no. 6055308) and spun the plates at 400×g in a swing-out rotor centrifuge for 20 sec to allow for cells to get attached to the glass. Cells were fixed in 1× PBS/4% paraformaldehyde (PFA) (Thermo Fisher Scientific, cat. no. 158127) for 15 min at room temperature and washed the cells with 1× PBS three times to remove the unreacted PFA. We permeabilized the cells in 1× PBS/0.5% saponin (Sigma-Aldrich, cat. no. 47036)/0.5% Triton X-100 (Sigma-Aldrich, cat. no. T8787) for 20 min at room temperature, followed by two washes in 1× PBS and then 15 min incubation in 0.1 N HCl at room temperature. We then washed the cells in 2× SSC at room temperature and incubated in them in 2× SSC/50% Formamide (Sigma-Aldrich, cat. no. F9037) for 20 min at room temperature. We precipitated 80 ng of each labelled BAC probe by adding 3 µg of COT-1 DNA (GeneON, cat. no. 3001), 20 µg of tRNA (Invitrogen cat. no. 10702487) and 2 volumes of 100% ethanol. Probes were centrifuged at 16,000×g for 20 min at 4 °C. We resuspended the DNA pellets in 30 µL of hybridization mix containing 2× SSC/50% formamide/10% dextran sulfate (Sigma-Aldrich, cat. no. 67578)/1% Tween-20 (Sigma-Aldrich, cat. no. P9416) and added 30 µL of each probe in hybridization mix to a single well of a 96-well plate. We performed denaturation performed at 85 °C for 10 min on a slide moat (Boekel Scientific, cat. no. 10630394), followed by plate spinning at 400×g for 20 sec. We then placed the plate in humidified chamber and

incubated overnight at 37 °C. The next day, we washed the wells three times with 1× SSC at 45 °C, 5 min each, followed by three consecutive washes with 0.1× SSC at 45 °C, 5 min each. Finally, we washed the wells once with 1× PBS at room temperature and stored the plate at 4 °C until imaging. We imaged the plates on the Opera Phenix high content screening confocal microscope (PerkinElmer) operated by the Harmony 4.8 software, using a 40× NA=0.8 water immersion lens (Olympus) and a 1.3 Megapixel CCD camera with pixel binning 2, corresponding to a pixel size of 299 nm. For each condition (transfection with KMT2A/HYLS1 sgRNAs or non-targeting sgRNA control), we imaged 50 fields with 11 planes in *z* per field in three technical triplicates per experiment and two biological experiments.

Calculation of the fraction of TK6-Cas9 edited cells. To calculate the percentage of TK6-Cas9 cells in which the expected 7 Mb deletion between *KMT2A* and *HYLS1* had occurred, we first segmented nuclei in the DNA FISH images based on the fluorescence background signal in maximally projected images, using a custom-made pipeline built in the Harmony software. We performed spot detection of all four colored FISH spots in different channels using a built-in analysis block in the Harmony software. To determine 3D distances between spots, we calculated the Euclidean spot-to-spot distances from the position of spots in maximally projected images in *x*,*y* and the distances between the *z*-planes of their brightest pixels. We corrected the distances between the *z* planes of spots in different colors for shifts due to chromatic aberration in *z*, by determining the offset of spot detection between the channels in *z* for one genomic locus simultaneously stained with all four different colored probes. We performed all the calculations using custom made R scripts (available upon request) with text files of analyses derived from the Harmony software as input. We called cells as harboring the 7 Mb deletion when the KMT2A-3′ and HYLS1-5′ probes (see scheme in **Supplementary Fig. 2a**) were not detected, and the distance between the KMT2A-5′ and HYLS1-3′ probes was smaller than a threshold set based on their average distance in non-targeted control cells. Similarly, we called cells as harboring the deletion plus chromosome 11 arm loss, when all probes were detected only once per cell, except the KMT2A-5′, which was detected twice. Finally, we called cells as harboring the deletion plus an amplification of 3′ arm of chromosome 11, when cells had two KMT2A-5′ probes, one KMT2A-3′ probe, one HYLS1-5′ probe and three HYLS1-3′ probes.

Targeted DNA sequencing

To profile mutations in cancer-associated genes in each region in the P3 and P6 prostate samples, we prepared libraries from 40 ng of gDNA per region using the TruSight Oncology 500 panel (Illumina, cat. no. 20040765). This panel is 1.94 Mb in size, encompassing the full coding sequencing of 523 cancer-related genes (coding size: 1.2 Mb). We sonicated 80 ng of genomic DNA extracted from each region in P3 and P6 samples using a Covaris Focused-ultrasonicator (Covaris) and then prepared libraries and performed two rounds of hybridization-based target capture following the manufacturer's instructions. We sequenced all the libraries on Illumina NovaSeq 6000 aiming at reaching minimum 500X read depth. We processed raw data using the TruSight Oncology 500 v2.2 Local App (Illumina) to generate fastq files by aligning the reads to the human reference sequence GRCh37 (hg19). We used the same application to perform QC and somatic variant calling using the tumor-only pipeline.

Whole genome sequencing

To profile germline CNVs in the P2 and P5 prostate samples, we extracted gDNA from peripheral blood using the DNeasy Blood & Tissue Kit (Qiagen, cat. no. 69504). To profile bulk SCNAs in two regions of the P5 prostate sample, we extracted the gDNA from the leftover nuclei suspension after single-nucleus sorting using the DNeasy Blood & Tissue Kit as above. We prepared individual libraries from each of the gDNA samples using the NEBNext Ultra II FS DNA Library Prep Kit (New England Biolabs, cat. no. E7805L) following the manufacturer's instructions. In brief, we used 50 ng of gDNA from each sample as input. We enzymatically fragmented the gDNA at 37 °C for 30 min followed by heat inactivation at 65 °C for 30 min to achieve a target size of approximately 200 bp. Subsequently, we performed end-repair and adapter ligation in the same tube followed by purification of the fragments using a 0.8 v/v ratio of Ampure XP beads (Beckman Coulter, cat. no. A63881). We amplified adapter-ligated DNA fragments with 5 PCR cycles with barcoded primers (New England Biolabs, cat. no. E7500) and purified the PCR product with a 0.9 v/v ratio of Ampure XP beads. We sequenced the peripheral blood gDNA libraries on NextSeq 2000 (Illumina) with pair-end mode using the NextSeq 1000/2000 P3 Reagents (300 Cycles) kit (Illumina, cat. no. 20040561), while the tissue block libraries were sequenced with single-end mode using the NextSeq 1000/2000 P2 Reagents (100 Cycles) kit (Illumina, cat. no. 20046811). See **Supplementary Data 7** for a summary of sequencing statistics.

3. Supplementary Tables

Supplementary Table 1. Pathological characteristics of the prostate samples used in this study.

Sample ID	Initial PSA (ng/mL)	Prostate volume (cm^3)	Tumor length (mm)
P1	6.9	33	44
P2	5.7	25	15
P3	5.8	51	24
P4	9	67	38
P5	11	53	35
P6	5.5	35	40
	**Pre-operative**		
**Sample ID**	**Stage**	**Gleason grading**	**Gleason score**
P1	cT1cN0M0	4+3	7b
P2	cT1cN0M0	3+4	7
P3	cT1cN0M0	3+4	7
P4	cT2N0M0	3+4	7
P5	cT1cN0M0	3+4	7
P6	cT2N0M0	4+3	7b
	**Post-operative**		
**Sample ID**	**Stage**	**Gleason grading**	**Gleason score**
P1	pT2c	3+4	7
P2	pT2c	3+4	7
P3	pT2c	3+4	7
P4	pT2c	3+4	7
P5	pT2c	3+4	7
P6	pT3a	4+3	7b
**Sample ID**	**Surgical margins**		
P1	Positive, 0.5 mm		
P2	Positive, 1 mm		
P3	Negative		
P4	Negative		
P5	Negative		
P6	Negative		

Supplementary Table 2. Characteristics of the donors of the brain and skeletal muscle samples profiled by scCUTseq in this study.

Sample ID*	Sex	Cause of death	Related conditions
ND428	M	Seizures	Alcoholism
ND436	F	Seizures	Epilepsy

*See **Supplementary Data 7** for a description of each library.

4. Supplementary References

1. Schneider, M. P. *et al. scAbsolute: measuring single-cell ploidy and replication status*. http://biorxiv.org/lookup/doi/10.1101/2022.11.14.516440 (2022) doi:10.1101/2022.11.14.516440.
2. Kaufmann, T. L. *et al.* MEDICC2: whole-genome doubling aware copy-number phylogenies for cancer evolution. *Genome Biol.* **23**, 241 (2022).
3. Kim, D., Paggi, J. M., Park, C., Bennett, C. & Salzberg, S. L. Graph-based genome alignment and genotyping with HISAT2 and HISAT-genotype. *Nat. Biotechnol.* **37**, 907–915 (2019).
4. Liao, Y., Smyth, G. K. & Shi, W. The R package Rsubread is easier, faster, cheaper and better for alignment and quantification of RNA sequencing reads. *Nucleic Acids Res.* **47**, e47 (2019).
5. Robinson, M. D., McCarthy, D. J. & Smyth, G. K. edgeR: a Bioconductor package for differential expression analysis of digital gene expression data. *Bioinforma. Oxf. Engl.* **26**, 139–140 (2010).
6. Gaujoux, R. & Seoighe, C. A flexible R package for nonnegative matrix factorization. *BMC Bioinformatics* **11**, 367 (2010).
7. Subramanian, A. *et al.* Gene set enrichment analysis: a knowledge-based approach for interpreting genome-wide expression profiles. *Proc. Natl. Acad. Sci. U. S. A.* **102**, 15545–15550 (2005).
