# Supplementary material for: High clonal diversity and spatial genetic admixture in early prostate cancer and surrounding normal tissue
Source: Nat Commun. 2024 Apr 24;15:3475. doi: 10.1038/s41467-024-47664-z (PMC11043350; doi:10.1038/s41467-024-47664-z)
Supplement: Supplementary file 3 — Description of Additional Supplementary Files [file 41467_2024_47664_MOESM3_ESM.pdf]

<u>Description of Additional Supplementary Files</u>

Supplementary Data Legends

Supplementary Data 1: Concentration and integrity of the RNA extracted from the regions in prostate samples P2 and P5 profiled by scCUTseq.

Supplementary Data 2: Concentration and integrity of the RNA extracted from the regions in prostate samples P2 and P5 profiled by scCUTseq.

Supplementary Data 3: Sequence of the oligos composing the DNA FISH probes used for scCUTseq validation.

Supplementary Data 4: Frequency of amplification or deletion of COSMIC genes in TRR/FER-specific and in the five most localized pseudo-diploid subclones in prostate samples P2 and P5.

Supplementary Data 5: List of genes mutated in prostate samples P2 and P5.

Supplementary Data 6: Sequence of the CUTseq oligonucleotide adapters used in this study.

Supplementary Data 7: Summary of sequencing runs.
