# Supplementary material for: High clonal diversity and spatial genetic admixture in early prostate cancer and surrounding normal tissue
Source: Nat Commun. 2024 Apr 24;15:3475. doi: 10.1038/s41467-024-47664-z (PMC11043350; doi:10.1038/s41467-024-47664-z)
Supplement: Supplementary file 11 — Reporting Summary [file 41467_2024_47664_MOESM11_ESM.pdf]

nature portfolio

Corresponding author(s): Nicola Crosetto

Last updated by author(s): Mar 25, 2024

Reporting Summary

Nature Portfolio wishes to improve the reproducibility of the work that we publish. This form provides structure for consistency and transparency in reporting. For further information on Nature Portfolio policies, see our Editorial Policies and the Editorial Policy Checklist.

Statistics

For all statistical analyses, confirm that the following items are present in the figure legend, table legend, main text, or Methods section.

n/a	Confirmed	
☐	☒	The exact sample size (*n*) for each experimental group/condition, given as a discrete number and unit of measurement
☐	☒	A statement on whether measurements were taken from distinct samples or whether the same sample was measured repeatedly
☐	☒	The statistical test(s) used AND whether they are one- or two-sided<br>*Only common tests should be described solely by name; describe more complex techniques in the Methods section.*
☒	☐	A description of all covariates tested
☒	☐	A description of any assumptions or corrections, such as tests of normality and adjustment for multiple comparisons
☐	☒	A full description of the statistical parameters including central tendency (e.g. means) or other basic estimates (e.g. regression coefficient) AND variation (e.g. standard deviation) or associated estimates of uncertainty (e.g. confidence intervals)
☐	☒	For null hypothesis testing, the test statistic (e.g. *F*, *t*, *r*) with confidence intervals, effect sizes, degrees of freedom and *P* value noted<br>*Give P values as exact values whenever suitable.*
☒	☐	For Bayesian analysis, information on the choice of priors and Markov chain Monte Carlo settings
☒	☐	For hierarchical and complex designs, identification of the appropriate level for tests and full reporting of outcomes
☐	☒	Estimates of effect sizes (e.g. Cohen's *d*, Pearson's *r*), indicating how they were calculated

Our web collection on statistics for biologists contains articles on many of the points above.

Software and code

Policy information about availability of computer code

Data collection

No software was used for data collection of the cell lines, prostate, breast, brain, and skeletal muscle samples. Data originating from The Cancer Genome Atlas were downloaded from cBioportal using the TCGAbiolinks (version 2.28.2) R package.

Data analysis

All code and versions of tools and pacakges used for data processing and analysis is also available at https://github.com/BiCroLab/scCUTseq

Sequencing data pre-processing
Raw sequence reads were basecalled and converted to fastq files using the BaseSpace Sequence Hub cloud service of Illumina. In the case of scCUTseq, each fastq file typically contains 384 single cells. We further demultiplexed each fastq file using a custom Python script. In short, we extracted cell-specific barcodes and UMIs from each read at the specified nucleotide locations. Following this, we matched the barcodes to a list of predefined barcodes allowing for two mismatches if using a set of 384 adapters, or one mismatch if using a set of 96 adapters. We then re-wrote the reads to cell-specific fastq files with the barcode and UMI appended to the read name. For sequence reads that extended through adapters, we trimmed the adapters from cell- (or sample-) specific fastq files using fastp (version 0.20.1). We aligned the fastq files to the hg19 human reference genome using bwa-mem (version 0.7.17-r1188) or to the dm6 Drosophila melanogaster reference in the case of S2 cells, and subsequently sorted and indexed using samtools (version 1.10). We moved barcodes and UMI tags from the bam file read header to the tags using a custom Python script. Finally, we deduplicated the reads based on the UMI tag and read position using umi-tools (version 1.1.1). In the case of WGS, we skipped the additional demultiplexing and trimming steps and deduplicated the reads using gatk MarkDuplicates (version 4.2.0.0) instead. All the procedures described above are fully automated and streamlined using snakemake (version 5.30.1).

Copy number calling
We counted reads in genomic bins of variable length (average length: 100 kb for bulk CUTseq; 250 kb for TK6 scCUTseq; 500 kb for all other

scCUTseq and ACT datasets) based on mappability. Genomic bins that are located in low mappability regions are extended, while bins in high mappability regions are shortened. Following this, we filtered out blacklisted regions, including telomeric and centromeric regions, using a blacklist adapted from https://github.com/Boyle-Lab/Blacklist. The adapted blacklist is available at https://github.com/BiCroLab/scCUTseq/ under snakemake pipelines. We then normalized reads for library size and GC-content. Briefly, we calculated the ratio between read counts in each bin and the mean read counts across all bins and log-normalized the ratio. For each bin, we computed the GC-content and modeled a weighted linear regression between the GC-content and the normalized read counts using the LOWESS R function. We used this model to scale the read counts, normalizing for GC-content. Next, we either segmented the normalized read counts using the Circular Binary Segmentation module in the DNAcopy (version 1.66.0) R package or, in the case of all single-cell breast and prostate tumor data, joint segmentation using the multipcf function in the copynumber (version 1.29.0.9) R package. Following this, we merged adjacent segments that were not significantly distinct using mergeLevels in the aCGH (version 1.78.0) R package. In the case of single-cell data, we then inferred integer copy numbers using a grid search between different ploidy (ranging from 1.7 to 6, using 0.01 step sizes) and purity (being 1 for single-cell data) combinations and selecting the combination with the lowest error. We skipped this last step in the case of bulk sequencing data.

Copy number quality control using a random forest classifier
To exclude low-quality single-cell copy number profiles from our analyses, we trained a random forest classifier54 based on 16 different copy number profile features (see Supplementary Data file 2), using the profiles of 2,304 single cells. In short, we manually annotated 2,304 scCUTseq profiles as high or low quality. We trained a random forest on 80% of these cells using the randomForest (version 4.6-14) R package with ntree = 500 and importance = TRUE ensuring class balance. To assess the performance of the random forest, we used the remaining 20% of the cells that were not used in the initial training as a validation set. Based on a receiver operating characteristic curve (ROC), we selected the most optimal threshold to classify single-cell copy number profiles.

Cell cycle analysis using scAbsolute
To assess whether some of copy number profiles discarded by our Random Forest classifier correspond to cycling cells in S phase, we applied the recently developed scAbsolute tool to infer the cycling activity of 991 cells from four different scCUTseq libraries (MS101 and MS102 from prostate sample P2 and NZ235 and NZ236 from prostate sample P5, see Supplementary Table 9Supplementary Data file 7). We ran scAbsolute using the workflow described here: https://github.com/markowetzlab/scDNAseq-workflow. After obtaining the cycling activity for each cell in those libraries, we predicted, for each library separately, whether the cells were in S-phase using the predict_replicating() function in scAbsolute.

Calculation of scCUTseq and ACT breadth of coverage and overdispersion
We first downsampled single cells to 800K reads. We then calculated the genome coverage using genomeCoverageBed from bedtools (version v2.25.0) and the overdispersion by calculating the variance of read counts per bin normalized by the mean read counts per bin.

Cell classification in tumor samples
We classified cells in three different groups (diploid, pseudo-diploid, and aneuploid) based on their copy number profile. To this end, we calculated the percentage of the genome that was altered in each cell, meaning non-diploid copy numbers in autosomes and a non-diploid copy number state in chrX for female samples and non-haploid chrX for male samples. In the case of prostate samples, we then classified all the cells with no alterations as diploid; cells with less than 25% of the genome altered as pseudo-diploid; and cells with more than 25% of their genome altered as aneuploid. For breast cancer samples, we did not make a distinction between pseudo-diploid and aneuploid cells but classified all the cells harboring copy number alterations as (potential) tumor cells.

Phylogenetic reconstruction and clone identification in tumor samples
We constructed phylogenetic trees of pseudo-diploid prostate cells and of tumor breast cells using MEDICC2 (version 0.8.1) with default parameters and total copy numbers of single cells as input. Following this, we used TreeCluster (version 1.0.3) to cluster cells based on the Newick tree generated by MEDICC2. We used the 'max' clustering method, which clusters leaves (cells) so that the maximum distance between leaves within the same cluster is at most t, with t equal to 3 and 4 for prostate samples P2 and P5, respectively, and 30 and 22 for breast cancer samples B1 and B2, respectively.

UMAP on aneuploid cell SCNA profiles
For aneuploid prostate cells, we first embedded the cells using UMAP with the following parameters: seed = 678, distance = 'manhattan', min_dist = 0 and n_neighbors = 6 (P2) and n_neighbors = 3 (P5). Subsequently, we clustered the cells using the hdbscan function in the dbscan (version 1.1-8) R package with minPts = 10.

Pseudo-diploid subclone spatial distribution
To quantify the spatial distribution of pseudo-diploid subclones in prostate samples, we calculated the Shannon entropy per clone as a proxy for how local or widespread clones are distributed. We normalized the number of cells from each clone based on the total number of cells that passed QC for each region and then calculated Shannon's entropy using the DescTools (version 0.99.49) R package. An entropy close to 0 indicates highly local distribution of a subclone while higher values indicate more widespread distributions.

TCGA Data analysis
On data obtained from TCGA, we classified patients in five different grade groups based on their Gleason score as following: 1) group 1: patients with 3+3 Gleason score; 2) group 2: patients with 3+4 Gleason score; 3) group 3: patients with 4+3 Gleason score; 4) group 4: patients with 4+4 Gleason score; and 5) group 5: patients with combined Gleason score 9 or 10. We then overlapped genomic regions that were either amplified, deleted or mutated with COSMIC and used ComplexHeatmap (version 2.16.0) to visualize the alterations. Finally, we used GISTIC262 (version 2.0.23) to look for genomic regions that are enriched for amplifications or deletions.

DNA FISH image analysis
We converted raw .nd2 image files to .tif format and performed 3D deconvolution using our in-house developed software Deconwolf using 100 iterations for all FISH channels and 50 iterations for the DNA channel. We reconstituted an overview of the entire tissue section by stitching the images acquired with 25x magnification, on which two pathologists independently manually annotated tumor and stroma regions using QuPath. In parallel and blindly from the tumor annotation, we manually quantified FISH dots in 50 images acquired at 100x magnification and deconvolved using ImageJ2.

For manuscripts utilizing custom algorithms or software that are central to the research but not yet described in published literature, software must be made available to editors and reviewers. We strongly encourage code deposition in a community repository (e.g. GitHub). See the Nature Portfolio guidelines for submitting code & software for further information.

Data

Policy information about availability of data

All manuscripts must include a data availability statement. This statement should provide the following information, where applicable:

- Accession codes, unique identifiers, or web links for publicly available datasets
- A description of any restrictions on data availability
- For clinical datasets or third party data, please ensure that the statement adheres to our policy

The sequencing data (raw) for all the cell lines and breast cancer samples have been deposited at ENA under accession code PRJEB71681 [https://www.ebi.ac.uk/ena/browser/view/PRJEB71681]. The sequencing data (raw) for prostate, brain, and skeletal muscle samples cannot be publicly shared, either because the ethical permit for collecting the samples explicitly excluded it (prostate) or because explicit informed consent was lacking (brain and skeletal muscle). However, these data may be shared with individual researchers after sending a formal request to the corresponding author, and only upon stipulation of a dedicated data transfer agreement, pending approval of the relevant ethical review board. An initial response to a request may be expected from the corresponding author within 2 weeks. All sequencing data sets have been aligned to the hg19 reference genome. The processed sequencing data for all samples (including the prostate, brain, and skeletal muscle), required to reproduce all analyses and recreate figures, are available as Source Data on figshare https://doi.org/10.6084/m9.figshare.23675517.v1.

Research involving human participants, their data, or biological material

Policy information about studies with human participants or human data. See also policy information about sex, gender (identity/presentation), and sexual orientation and race, ethnicity and racism.

Reporting on sex and gender	Sex and/or gender was determined based on self-report. All prostate cancer samples are from male patients aged 43-65 years old. Due to the nature of the disease (exclusively to the male sex) we did not further consider sex differences. Breast cancer samples are two female patients, aged 55–65, brain/skeletal muscle donors were one male and one female, aged 45–50. Since the breast cancer samples and brain/skeletal muscle samples were not used as a comparison to the prostate cancer (or each other) and purely for technical validation, we did not take the sex into account during the analysis.
Reporting on race, ethnicity, or other socially relevant groupings	We did not gather or have access to race, ethnicity or other socially relevant groupings and thus, we did not control for these factors.
Population characteristics	We did not study specific population characteristics, such as genotypic information or differential diagnosis/treatment categories. We performed our study on six samples from prostate cancer patients and did compare different population characteristics between these samples. Breast cancer and brain/skeletal muscle samples were used from a technical validation point and we also did not compare different population characteristics between the two breast cancer samples or the two brain/skeletal muscle samples.
Recruitment	Prostate samples were prospectively collected after obtaining written informed consent from patients with early localized prostate cancer. Prostate samples were selected based on the diagnosis of early localized prostate cancer and there was no self-selection.<br>Breast cancer samples were retrospectively collected from two Luminal B-like breast cancer specimens from patients who gave written informed consent.<br>The forebrain and skeletal muscle samples were procured through the Karolinska Institutet Donatum Tissue Collection program, which represents a collection of tissues from deceased individuals who donated their body for research purposes. The donors gave written informed consent for the usage of their tissues for other studies. Since this study was conceived after their death, ethical approval was given later to use these tissues for additional studies including ours.
Ethics oversight	All research complies with the ethical regulations and was approved by the relevant Ethical Committees. The collection of prostate cancer samples was approved by the Regional Ethics Committee of Sweden, ethical permit: 2018/1003-31. Collection of brain and skeletal muscle samples was approved by the Regional Ethics Committee of Sweden, ethical permit: 2010/313-31/3. Collection of breast cancer samples was approved by Comitato Etico dell'Istituto Oncologico di Candiolo IRCCS, ethical permit: "Profiling", 001-IRCC-00IIS-10.

Note that full information on the approval of the study protocol must also be provided in the manuscript.

Field-specific reporting

Please select the one below that is the best fit for your research. If you are not sure, read the appropriate sections before making your selection.

☒ Life sciences ☐ Behavioural & social sciences ☐ Ecological, evolutionary & environmental sciences

For a reference copy of the document with all sections, see nature.com/documents/nr-reporting-summary-flat.pdf

Life sciences study design

All studies must disclose on these points even when the disclosure is negative.

Sample size	No power calculation was performed. Sample size was determined by the availability of samples and feasibility of processing these samples

Sample size	(both logistically and economically). Since this is a proof-of-concept study, the current sample size of two fully profiled prostate cancer patients was large enough from a technical standpoint.
Data exclusions	From the seven prospectively collected prostatectomy samples, we excluded one sample due to very low DNA quality.
Replication	To verify reproducibility, all single-cell data is performed in batches of at least 96 cells per condition (for the prostate cancer samples thousands of cells total). All data was then compared in terms of reproducibility and similarity. All these comparisons have been reported in (supplementary) figures in the manuscript. The cell line mixture experiment was performed in two replicates of 96 cell each replicate, which were both successful.
Randomization	No randomization was performed since this is a proof of concept study on only two prostatectomy samples. Four other prostatectomy samples, 2 breast cancer samples, and 2 brain/skeletal muscle samples were used for technical validation. Due to the sample size, randomization was not possible or applicable.
Blinding	There was no blinding performed. Since this was a technical proof-of-concept study and we did not compare differential treatments, blinding was not applicable in this study.

Reporting for specific materials, systems and methods

We require information from authors about some types of materials, experimental systems and methods used in many studies. Here, indicate whether each material, system or method listed is relevant to your study. If you are not sure if a list item applies to your research, read the appropriate section before selecting a response.

Materials & experimental systems

n/a	Involved in the study
☒	☐ Antibodies
☐	☒ Eukaryotic cell lines
☒	☐ Palaeontology and archaeology
☒	☐ Animals and other organisms
☒	☐ Clinical data
☒	☐ Dual use research of concern
☒	☐ Plants

Methods

n/a	Involved in the study
☒	☐ ChIP-seq
☒	☐ Flow cytometry
☒	☐ MRI-based neuroimaging

Eukaryotic cell lines

Policy information about cell lines and Sex and Gender in Research

Cell line source(s)	IMR90, SKBR3 and MCF10A cell lines from the American Tissue Culture Collection (ATCC, cat. no. CCL-186, HTB-30, and CRL-10317, respectively) and Drosophila S2 cells from Gibco (cat. no. R69007). TK-6 cells as described by Gothe et al, Molecular Cell 2019.
Authentication	None of the cell lines used in this study were authenticated
Mycoplasma contamination	All cell lines tested negative for mycoplasma contamination using the MycoAlert Mycoplasma Detection kit (Lonza, cat. no. LT07-118).
Commonly misidentified lines (See ICLAC register)	None of the cell lines used in this study is registered in the International Cell Line Authentication Committee (ICLAC) database of misidentified cell lines

Plants

Seed stocks	NA, no plants were used in this study
Novel plant genotypes	NA, no plants were used in this study
Authentication	NA, no plants were used in this study
